# The domestication event of the Tibetan pig revealed to be in the upstream region of the Yellow River based on the mtDNA D-loop

**DOI:** 10.5713/ajas.19.0275

**Published:** 2019-07-01

**Authors:** Qianyun Ge, Caixia Gao, Yuan Cai, Ting Jiao, Jinqiang Quan, Yongbo Guo, Wangshan Zheng, Shengguo Zhao

**Affiliations:** 1 College of Animal Science and Technology, Gansu Agricultural University, Lanzhou 730070, China; 2 State Key Laboratory of Veterinary Biotechnology, Harbin Veterinary Research Institute, Chinese Academy of Agricultural Sciences, Harbin 150069, China; 3 College of Grassland, Gansu Agricultural University, Lanzhou 730070, China; 4 State Key Laboratory of Genetic Resources and Evolution, Kunming Institute of Zoology, Chinese Academy of Sciences, Kunming 650223, China

**Keywords:** Tibetan Pig, mtDNA D-Loop, Polymorphism, Phylogeny, Upstream Region of the Yellow River

## Abstract

**Objective:**

Evidence from previous reports indicates that pig domestication in East Asia mainly occurred in the Mekong region and the middle and downstream regions of the Yangtze River. Further research identified two new origin centers for domestic pigs in the Tibetan Plateau and the islands of Southeast Asia. However, due to the small sample size of Tibetan pigs, details of the origin and spread of Tibetan pigs has not yet been established.

**Methods:**

We analyzed mitochondrial DNA control region (D-loop) variation in 1,201 individuals from nine Tibetan pig populations across five provinces. Comprehensive Tibetan pig samples were taken to perform the most detailed analysis of Tibetan pigs to date.

**Results:**

The result indicate that Rkaze pigs had the lowest level of diversity, while Changdu pigs had the highest diversity. Interestingly, these two populations were both in the Tibetan Plateau area. If we calculate diversity in terms of each province, the Tibetan Plateau area had the lowest diversity, while the Chinese province of Gansu had the highest diversity. Diversity gradient analysis of major haplotypes suggested three domestication centers of Tibetan pigs in the Tibetan Plateau and the Chinese provinces of Gansu and Yunnan.

**Conclusion:**

We found two new domestication centers for Tibetan pigs. One is in the Chinese province of Gansu, which lies in the upstream region of the Yellow River, and the other is in the Chinese province of Yunnan.

## INTRODUCTION

Tibetan pigs are mainly distributed in the Tibetan Plateau area, where is the largest continuous high altitude ecosystem in the world with an average altitude of more than 4,000 m [1]. These animals have significant phenotypic and physiological differences compared to lowland pigs, allowing them to adapt to extreme conditions such as hypoxia [1]. Evidence from previous reports indicates that pig domestication in East Asia mainly occurred in the Mekong region and the middle and downstream regions of the Yangtze River [2]. Further studies using mtDNA hypervariable segment I variation in seven Tibetan pig populations identified two new origin centers for domestic pigs in the Tibetan highlands and the islands of Southeast Asia [1]. Domestic pig bones were discovered in the Tibetan Plateau area around 5,000 years old indicate that Tibetan people could domesticate the Tibetan pig which strongly supports that the Tibetan pig may have a local origin. There is evidence that the isolated Tibetan pleateu area have both the substantial genetic diversity and the highly divergent lineage, this discovery also support that the local domestication of the Tibetan pig [3]. However, due to the small sample size of Tibetan pigs, details of the origin and spread of Tibetan pigs has not yet been established. mtDNA sequence analysis has enabled important investigation of the origin and diversification of animal populations [4–8]. mtDNA contains the displacement (D)-loop, which contains regulatory sequences controlling both replication and transcription of mtDNA [9]. In the present study, the mtDNA control region (D-loop) from nine Tibetan populations across five provinces was used to identify the origin and dispersal of Tibetan pigs. In addition to 473 sequences retrieved from GenBank, 728 novel individuals were sampled from different regions where the samples had not been adequately collected before and we downloaded some domestic pig and wild boar sequences to compare with Tibetan pigs (Supplementary Table S1). Therefore, we provide the most comprehensive screening of mtDNA variations among Tibetan pigs to obtain genetic information on the evolutionary domestication history of the Tibetan pig.

## MATERIALS AND METHODS

All animal work was conducted according to the guidelines for the Institutional Animal Care and Use Committee (IACUC) and was approved by the Animal Care Committee of Gansu Agricultural University.

### Sampling and sequencing

Blood or tissue samples were collected from 728 Tibetan pigs (a list of collected and supplementary animals, with GenBank accession number and other detailed information, is provided in an editable format in Supplementary Table S1). DNA was extracted and purified using the phenol-chloroform extraction method [4]. A fragment of the mtDNA control region (D-loop) was amplified using the primers 5′-CCAAAAAC AAAGCAGAGTGTAC-3′ and 5′ -CGTTATGAGCTACCG TTATA-3′ [10]. The PCR mixture volume was 25 μL and contained 12.5 μL of 2× Eco Taq PCR Supermix containing 1 U Taq polymerase, 500 mμ dNTPs, and 10× Taq buffer (Beijing TransGen Biotech Co., Ltd., Beijing, China), 0.1 μg of template DNA, 0.4 μL of each primer at 10 pmol/mL and 11.6 μL of ddH2O. The cycling conditions were initial denaturation at 94°C for 5 min, followed by 33 cycles of 94°C for 30 s, 56°C for 30 s and 72°C for 30 s and a final extension for 5 min at 72°C [10]. Amplified DNA fragments were purified following agarose gel electrophoresis and sequenced using the ABI 3130 DNA sequencer (Applied Biosystems, Foster City, CA, USA) [10].

### Data analysis

To obtain high coverage of Tibetan pigs, the dataset of 473 samples was complemented with data on representative Tibetan pigs (Supplementary Table S1). Original sequence data were obtained using the ABI PRISM DNA sequencer software. Sequences were edited by the DNASTAR package and aligned using ClustalX 1.81 [11]. MEGA 7.0 was used to collect sequences and construct a phylogenetic tree [12]. DnaSP 5.0 software was used to analyze the haplotypes and genetic diversity [10]. The median-joining network plot of mtDNA control region sequences was constructed using the Network program. Correlation analysis and principal component analysis (PCA) were investigated by SPSS 19.0.

## RESULTS

### Genetic diversity analysis

The 431-bp D-loop region of mtDNA was used to analyze single nucleotide polymorphisms (SNPs) for all 1,201 sequences in Tibetan pigs (Diqing n = 178, Linzhi n = 241, Shannan n = 91, Changdu n = 90, Rkaze n = 24, Aba n = 70, Ganzi n = 133, Hezuo n = 268, Qinghai n = 106). We identified a total of 42 polymorphic sites (representing 9.7% of the total DNA sequence analyzed), including 13 singleton variable sites and 29 parsimony informative sites. One insertion/deletion (indel) was detected in our novel sequences. The transition:transversion ratio R (Ts/Tv) was 3.34, indicating a strong transitional bias that is common in mammalian mitochondrial evolution [13,14].

The genetic diversity of Tibetan pigs was calculated (Table 1). As shown in Table 1, the haplotype diversity (Hd) of Tibetan pigs ranged from 0.420 to 0.883. Nucleotide diversities (Pi) ranged from 0.00128 to 0.00435. The average number of nucleotide differences (K) was between 0.551 and 1.876. All three values of the Changdu pigs were the highest, and the values of the Rkaze pigs were the lowest among the nine populations. Interestingly, both populations were in the Tibetan Plateau area. If all samples were analyzed as a population, Hd was 0.812±0.008, Pi was 0.00368, and K was 1.584.

The correlation of Pi, Hd, and K was analyze d using SPSS 19.0 (Table 2). The results showed that Hd, Pi, and K were positively correlated with each other, which indicated that all three indexes influenced the abundant degree of genetic diversity. The PCA was used for the synthetic assessment of genetic diversity. The results are shown in Table 3. We obtained a synthesized assessment score (Fz). The Fz score indicated the highest genetic diversity in Changdu pigs and the lowest in Rkaze pigs.

### Haplotype analysis of sequences

In total, 60 haplotypes were identified according to the distribution of variable sites in all 1,201 Tibetan pig sequences (Supplementary Table S2). Distribution frequencies of haplotypes indicated no equilibrium. Thirty-eight haplotypes were exclusive, accounting for 63.3%. Shared haplotypes H4 and H46 (T) emerged in all nine populations; H4 had the highest frequency rate, at 36.5% (438/1,201). H46 (T) had the second highest, at 16.3%.

## DISCUSSION

### Genetic diversity of Tibetan pigs

Hd, Pi, and K are the basic parameters used to assess genetic diversity. Hd is a measure of the uniqueness of a particular haplotype in a given population, which reflects haplotype abundance in a population [15]. Pi and K measure the degree of intrapopulation haplotype mutation [16]. Tibetan pigs had high Hd and low Pi values (Table 1), indicating that Tibetan pigs may grow rapidly after a bottleneck or founder event effect by a small effective population, although the mutation leads to the accumulation of Hd, but nucleotide diversity has not yet accumulated [17]. Under the assumptions of selective neutrality and population balance, Tajima’s D and Fu’s Fs test values tend to be negative under an excess of recent mutations, which is considered to be evidence of population growth [15,16]. Tibetan pigs all revealed negative Tajima’s D and Fu’s Fs test values (not including Rkaze and Aba), suggesting population expansion in the past (Table 1).

The PCA is a statistical procedure used to reduce the di mensionality of a dataset by transformation to a new set of variables (the principal components) to summarize the features of the data [17]. The diversity parameters, including Hd, Pi, and K, were analyzed using PCA. We obtained the Fz (Table 3). The Fz ranged from −3.324 to 1.993, and its rank indicated that Rkaze pigs had the lowest level of diversity, while Changdu pigs had the highest diversity. These two populations are all in the Tibetan Plateau area. If we calculate the PAC in terms of each province (Table 3), the Tibetan Plateau area had the lowest diversity, whereas the Gansu Province, which lies in the upstream region of the Yellow River, had the highest diversity. Genetic diversity is essential for continued reproduction. Tibetan pigs, as China’s unique and excellent species, have a high status of genetic diversity in domestic pigs, but they also face serious threats from exotic pig breeds, which in turn affect the development and utilization of the outstanding advantages of plateau hypoxia adaptability, so the protection of the Tibetan pig from extinction is important.

### Phylogenetic analysis of Tibetan pigs

The first systematic study of mtDNA polymorphisms in wild boar and domestic pig populations is almost universal, and results show that at least six different centers have domesticated pigs [7]. Previous studies have shown that the geographically isolated Tibetan pleateu have both the substantial genetic diversity and the highly divergent lineage, which strongly support the local domestication of the Tibetan pig [3]. For more details the origin and spread of Tibetan pigs, the present study comprehensively analyzed the mtDNA data of Tibetan pigs and provided valuable insights into Tibetan pig phylogenetic analysis. The mtDNA D-loop region (431 bp) of 728 Tibetan pigs was determined with the dataset of 473 samples were complemented. The 9 D-loop region sequences of other wild boars and domestic pigs (Supplementary Table S1), each representing a unique haplotype in their population, were selected from Larson et al [7] paper and downloaded from GenBank as references. While the choice of specific representative samples in a certain population was randomly selected, each sample from the same population has equal potential to allow us to determine the schematic backbone and mutation motif of this population [18]. We can clarify the origin of this breed and provide a theoretical basis for the degree and pattern of genetic material introgress Tibetan pigs from exotic breeds by using a comparative mtDNA analysis of the Tibetan pig with relevant populations [1].

**Figure 1:**
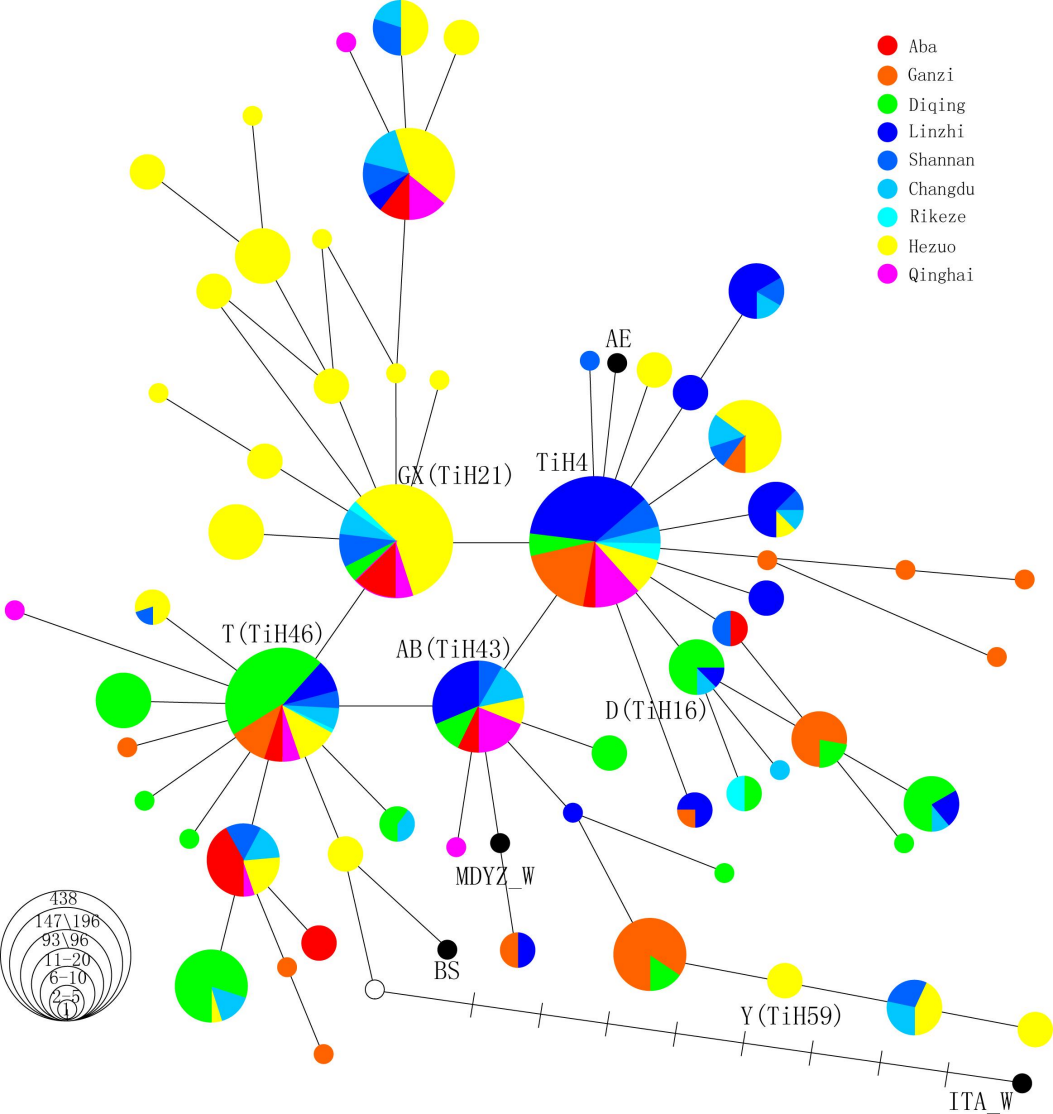
The median network of Tibetan pig and other pig mitochondrial DNA (mtDNA) based on the sequence variation in the control region. The mtDNA control region fragment covers the region from 15,372 to 15,414 relative to the AF486859 reference sequence. These samples are from the nine populations of Tibetan pigs in five provinces, including Linzhi, Shannan, Changdu, and Rkaze pigs from the Tibetan Plateau area; Aba and Ganzi pigs from Sichuan Province; Hezuo pigs from Gansu Province; Diqing pigs from Yunnan Province; Qinghai pigs from Qinghai Province; and other pig breeds. Each haplotype is represented by a circle, with the area of the circle proportional to its frequency. Samples from different regions are indicated by different colors. The length of each branch is proportional to the number of mutations on the respective branch. In parentheses are the haplotypes we named, which are the same as the reference haplotype in Larson’s article.

The median-joining network method was used to construct a haplotype network structure. The regional distribution of the main founders belonging to these haplotypes were depicted in the network (Figure 1). The haplotype network (Figure 1) emphasizes that the four core haplotypes (TiH4, GX, T, AB) form a parallelogram of a mutation length and a considerable derivatives can be detected in Tibetan pigs. These haplotypes demonstrated a perfect star-like profile that was typical of dramatic population growth.

Clade TiH4 exhibited a strongly star-like profile, harbor ing the most haplotypes, which best shows the full extent of the diversity among the four major clades. The core haplotype TiH4 was represented by samples taken from all nine examined populations and harbors the most individuals identified in this study. More than half of the Tibetan pigs in this haplotype were mainly from the Tibetan Plateau area, indicating that this clade was older than the others and domestication of Tibetan pigs mainly occurred in the Tibetan Plateau area. Consistent with a previous study, both the substantial genetic diversity and the highly differentiated lineage in Tibetan Plateau support the local domestication of the Tibetan pig [1].

Clade T also presents a star-like profile. Its core haplotype T is also widely distributed in all nine populations of Tibetan pigs, but its frequency rate lower than that of TiH4. Diqing pigs (distributed in the Chinese province of Yunnan) were mainly found in haplotype T, indicating that this clade was mainly domesticated in Yunnan Province, demonstrating this province was one of the domestication centers of Tibetan pigs. A previous study showed that the high diversity found in Yunnan province might be due to its location at the crossroads between the three different domestication centers of the middle and downstream regions of the Yangtze River, Southeast Asia, and Tibetan Plateau area [1]. This study specified that a small domestication event of Tibetan pigs may have occurred in the Yunnan Province.

Meanwhile, clade AB and clade GX were extended from clade T. Clade AB was mainly made up of Tibetan pigs from the Tibetan Plateau area, and clade GX was made up of Hezuo pigs distributed in the Chinese province of Gansu. These two areas were located north of the Yunnan Province. Indicated that the domestication migrated north from the Mekong region and the islands of Southeast Asia. The trade between Tibet and southwestern China was carried out through the so-called Tea-horse Ancient Road, including the Yunnan-Tibet and the Sichuan-Tibet routes, understandably, in most cases, the distribution of domestic animals may follow the migration of their owners. The Tibetans pigs in clade AB from Yunnan province into the Tibetan highlands might via the Tea-horse Ancient Road. In addition, clade AB connected with the wild boar of the middle and downstream region of the Yangtze River, which was regarded as one of the origin and dispersal centers of cultivated rice and a center of agriculture civilization in East Asia [19]. And there was a Tangfan Ancient Road between Chang’an and Lhasa. Tibetan pigs of clade GX were distributed into Gansu Province might via the Tangfan Ancient Road.

The diversity gradient determined by the number of de rived haplotypes, the number of unique haplotypes and the proportionate allocation to the core haplotypes may have value in furthering our understanding of the origin of this clade [20]. The present mtDNA data show a gradient in the diversity of clade GX. This strain possesses many one-, two-, or greater-than-two-mutation distance derivatives detectable in Tibetan pigs and exhibits a perfect star-like profile. The number of unique haplotypes is the greatest in clade GX, which was mainly made up of Hezuo pigs (distributed in the Chinese province of Gansu in the upstream region of the Yellow River), and derivative haplotypes were also limited in Hezuo pigs, revealing that domestication episodes were concentrated in Gansu Province but later than in the Tibetan Plateau area. A small star-like profile implicated there was also happened a small-scale secondary domestication. Larson et al [7] showed that GX found in wild boar (morphologically wild individuals from the Chinese province of Gansu) is shared by several East Asian domestic pigs. A previous study has also reported that there was a haplogroup consisted of an independent clade of Tibetan pigs from the highlands in the upstream region of the Yellow River that was restricted to Hezuo pig [1]. Evidence from ancient DNA also proves that a domestication episode appeared in this area. Gansu Province was revealed to be a potential diversity center, and this study showed the domestication of Tibetan pigs in the upstream region of the Yellow River for the first time.

### The major clades in the phylogenetic tree of Tibetan pigs

**Figure 2:**
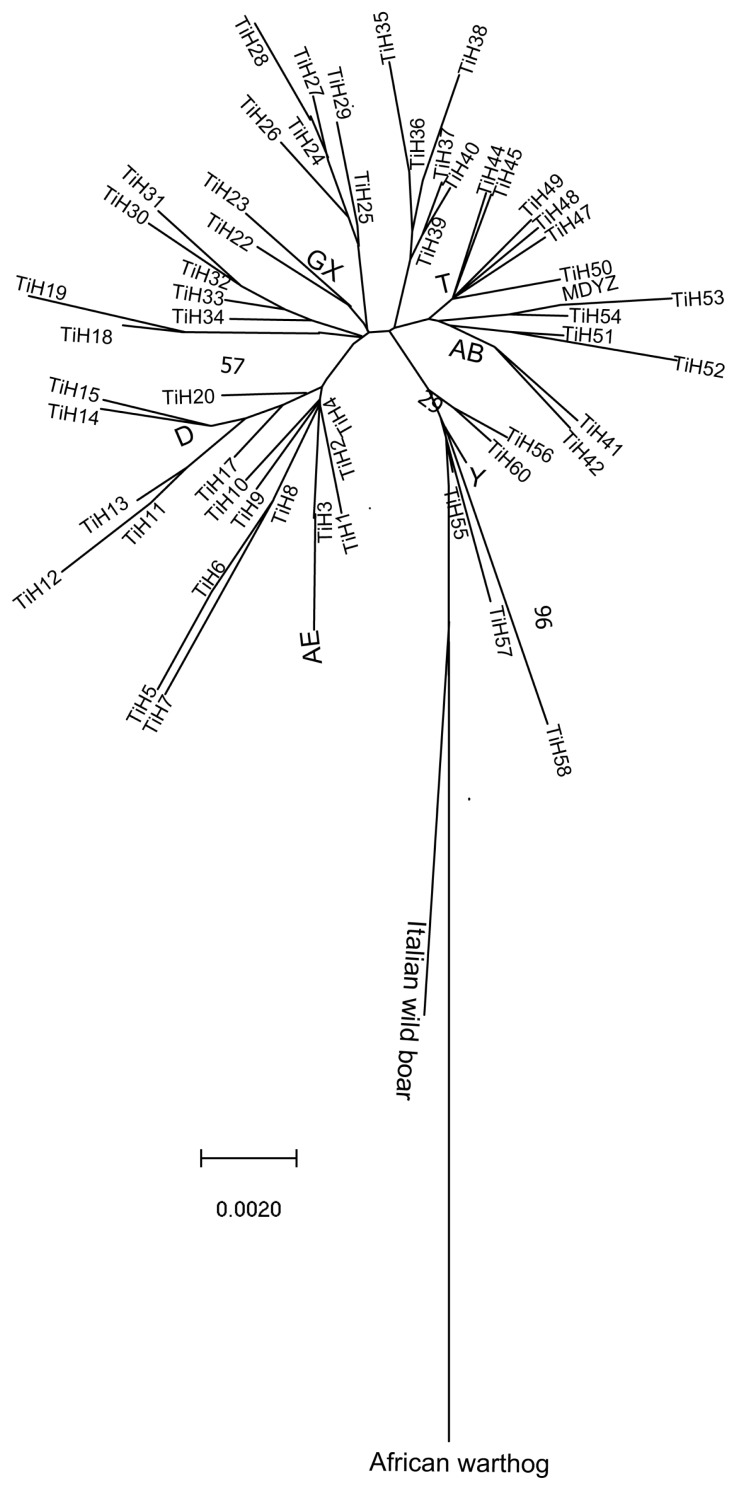
The phylogenetic tree of Tibetan pigs and other pig breeds based on DNA control region (D-loop) sequences. The tree was constructed by the neighbor-joining (NJ) method with the African warthog as the outgroup. The evolutionary history was inferred using the NJ method [21]. The optimal tree with the sum of branch length = 0.30134799 is shown. The percentage of replicate trees in which the associated taxa clustered together in the bootstrap test (1,000 replicates) are shown next to the branches [22]. The tree is drawn to scale, with branch lengths in the same units as those of the evolutionary distances used to infer the phylogenetic tree. The evolutionary distances were computed using the Kimura 2-parameter method [23] and are in units of the number of base substitutions per site. The rate variation among sites was modeled with a gamma distribution (shape parameter = 1). The analysis involved 69 nucleotide sequences. The codon positions included were 1st+2nd+3rd+Noncoding. All positions containing gaps and missing data were eliminated. There were a total of 428 positions in the final dataset. Evolutionary analyses were conducted in MEGA7 [12].

The information gleaned from the mtDNA genome tree enabled us to conduct a phylogenomic analysis for Tibetan pigs. The African warthog is distinct from Eurasian wild boars and has frequently been used as the outgroup in previous phylogenetic studies of pigs [14]. In the present study, we used a mtDNA D-loop region from the African warthog as the outgroup to root the mtDNA genome tree. Sixty Tibetan pig haplotypes (Table 2) and 9 selected D-loop sequences of other pig breeds (Supplementary Table S1) were used to construct a phylogenetic tree based on pairwise genetic distances by the neighbor-joining (NJ) method (Figure 2). A preliminary phylogenetic analysis of these D-loop sequences was performed and revealed several clades in the tree. As seen from the picture (Figure 2), the NJ tree of 69 mtDNA genomes (including 9 selected D-loop sequences of other pig breeds) revealed two major clades. All Tibetan pigs were clustered together distinct from Italian wild boar that forms a single clade. The structure of the phylogenetic tree is the same as the network. All Tibetan pigs revealed four major clades in the tree, corresponding to four clades in the network (clade H4, clade T, clade GX, and clade AB).

## CONCLUSION

In the present study, the use of genetic diversity, network and phylogenetic tree of control region mtDNA sequences allowed us to conduct a phylogeographic analysis of Tibetan pigs. This approach could also be utilized to elucidate the origin of other domestic animals. Our findings indicate that the Chinese provinces of Gansu (located in the upstream region of the Yellow River) and Yunnan might be another two domestication centers of Tibetan pigs.

## Supplementary Data



## Figures and Tables

**Table 1:** Parameters for determination of genetic diversity of Tibetan pigs

Population	Size	S	h	Hd1)	Pi	K	Fu’s F	Tajima’s D
Aba pig	70	6	8	0.839±0.018	0.00363	1.563	−0.827	0.60968
Ganzi pig	133	15	14	0.596±0.044	0.00336	1.449	−5.274	−1.26021
Diqing pig	178	12	16	0.717±0.032	0.00338	1.458	−6.611	−0.74094
Linzhi pig	241	12	14	0.538±0.036	0.00200	0.860	−8.147	−1.34760
Shannan pig	91	13	14	0.816±0.029	0.00354	1.525	−5.893	−1.10357
Changdu pig	90	16	17	0.883±0.014	0.00435	1.876	−7.670	−1.15051
Rkaze pig	24	2	3	0.420±0.110	0.00128	0.551	0.102	0.06219
Hezuo pig	268	20	27	0.845±0.015	0.00409	1.763	−16.692	−1.18627
Qinghai pig	106	11	10	0.725±0.036	0.00317	1.380	−2.313	−0.88850
Total	1,201	42	60	0.812±0.008	0.00368	1.584	−66.116	−1.82904

S, number of polymorphic (segregating) sites; Hd, number of haplotypes; Pi, nucleotide diversity, Nei 1987, eqs. 10.5 or 10.6 (Masatoshi Nei); K, average number of nucleotide differences, Tajima 1983, eq. A3 (Tajima).

1) Hd±SD, haplotype (gene) diversity and sampling variance, Nei 1987, eqs. 8.4 and 8.12 but replacing 2n with n. The standard deviation (or standard error) is the square root of the variance (Masatoshi Nei) [8].

**Table 2:** Correlation matrix between indexes

Item	Hd	Pi	K
Hd	1.000	0.931	0.931
Pi	0.931	1.000	1.000
k	0.931	1.000	1.000

Hd, number of haplotypes; Pi, nucleotide diversity; K, average number of nucleotide differences.

**Table 3:** Rank and general scores of principal components of different populations

Province	Fz	Rank	Population	Fz	Rank
Sichuan	0.381	2	Aba pig	0.974	3
			Ganzi pig	−0.212	7
Yunnan	0.244	3	Diqing pig	0.244	5
Tibet	−0.648	5	Linzhi pig	−2.046	8
			Shannan pig	0.785	4
			Changdu pig	1.993	1
			Rkaze pig	−3.324	9
Gansu	1.546	1	Hezuo pig	1.546	2
Qinghai	0.039	4	Qinghai pig	0.039	6

Fz, synthesized assessment score.
