# Supplementary material for: The domestication event of the Tibetan pig revealed to be in the upstream region of the Yellow River based on the mtDNA D-loop
Source: Asian-Australas J Anim Sci. 2019 Jul 1;33(4):531–8. doi: 10.5713/ajas.19.0275 (PMC7054604; doi:10.5713/ajas.19.0275)
Supplement: Supplementary file 1 [file ajas-19-0275-suppl.pdf]

Supplementary Table S1. Information on sampling.

Sample ID	Accession	Breed/status	Location	References
AT001	EF545578	Aba Tibetan pig	Sichuan,Aba	Gui-Sheng Wu et al.2007
AT002	DQ496269	Aba Tibetan pig	Sichuan,Aba	Gui-Sheng Wu et al.2007
AT003	DQ496273	Aba Tibetan pig	Sichuan,Aba	Gui-Sheng Wu et al.2007
AT004	DQ496272	Aba Tibetan pig	Sichuan,Aba	Gui-Sheng Wu et al.2007
AT005	DQ496271	Aba Tibetan pig	Sichuan,Aba	Gui-Sheng Wu et al.2007
AT006	DQ496270	Aba Tibetan pig	Sichuan,Aba	Gui-Sheng Wu et al.2007
AT007	DQ496268	Aba Tibetan pig	Sichuan,Aba	Gui-Sheng Wu et al.2007
AT008	DQ496267	Aba Tibetan pig	Sichuan,Aba	Gui-Sheng Wu et al.2007
AT009	DQ496266	Aba Tibetan pig	Sichuan,Aba	Gui-Sheng Wu et al.2007
AT010	DQ496263	Aba Tibetan pig	Sichuan,Aba	Gui-Sheng Wu et al.2007
AT011	DQ496262	Aba Tibetan pig	Sichuan,Aba	Gui-Sheng Wu et al.2007
AT012	DQ496261	Aba Tibetan pig	Sichuan,Aba	Gui-Sheng Wu et al.2007
AT013	DQ496260	Aba Tibetan pig	Sichuan,Aba	Gui-Sheng Wu et al.2007
AT014	DQ496259	Aba Tibetan pig	Sichuan,Aba	Gui-Sheng Wu et al.2007
AT015	DQ496258	Aba Tibetan pig	Sichuan,Aba	Gui-Sheng Wu et al.2007
AT016	DQ496257	Aba Tibetan pig	Sichuan,Aba	Gui-Sheng Wu et al.2007
AT017	DQ496256	Aba Tibetan pig	Sichuan,Aba	Gui-Sheng Wu et al.2007
AT018	DQ496255	Aba Tibetan pig	Sichuan,Aba	Gui-Sheng Wu et al.2007
AT019	DQ496254	Aba Tibetan pig	Sichuan,Aba	Gui-Sheng Wu et al.2007
AT020	DQ496253	Aba Tibetan pig	Sichuan,Aba	Gui-Sheng Wu et al.2007
AT021	DQ496252	Aba Tibetan pig	Sichuan,Aba	Gui-Sheng Wu et al.2007
AT022	DQ496251	Aba Tibetan pig	Sichuan,Aba	Gui-Sheng Wu et al.2007
AT023	HQ148355	Aba Tibetan pig	Sichuan,Aba	Shuli Yang et al.2011
AT024	HQ148354	Aba Tibetan pig	Sichuan,Aba	Shuli Yang et al.2011
AT025	HQ148353	Aba Tibetan pig	Sichuan,Aba	Shuli Yang et al.2011
AT026	HQ148352	Aba Tibetan pig	Sichuan,Aba	Shuli Yang et al.2011
AT027	HQ148351	Aba Tibetan pig	Sichuan,Aba	Shuli Yang et al.2011
AT028	HQ148350	Aba Tibetan pig	Sichuan,Aba	Shuli Yang et al.2011
AT029	HQ148349	Aba Tibetan pig	Sichuan,Aba	Shuli Yang et al.2011
AT030	HQ148348	Aba Tibetan pig	Sichuan,Aba	Shuli Yang et al.2011
AT031	HQ148347	Aba Tibetan pig	Sichuan,Aba	Shuli Yang et al.2011
AT032	HQ148346	Aba Tibetan pig	Sichuan,Aba	Shuli Yang et al.2011
AT033	HQ148345	Aba Tibetan pig	Sichuan,Aba	Shuli Yang et al.2011
AT034	HQ148344	Aba Tibetan pig	Sichuan,Aba	Shuli Yang et al.2011
AT035	JX068390	Aba Tibetan pig	Sichuan,Aba	Jin,L et al.2013
AT036	JX068389	Aba Tibetan pig	Sichuan,Aba	Jin,L et al.2013
AT037	JX068388	Aba Tibetan pig	Sichuan,Aba	Jin,L et al.2013
AT038	JX068387	Aba Tibetan pig	Sichuan,Aba	Jin,L et al.2013
AT039	JX068386	Aba Tibetan pig	Sichuan,Aba	Jin,L et al.2013
AT040	JX068385	Aba Tibetan pig	Sichuan,Aba	Jin,L et al.2013
AT041	JX068384	Aba Tibetan pig	Sichuan,Aba	Jin,L et al.2013
AT042	JX068383	Aba Tibetan pig	Sichuan,Aba	Jin,L et al.2013
AT043	JX068382	Aba Tibetan pig	Sichuan,Aba	Jin,L et al.2013
AT044	JX068381	Aba Tibetan pig	Sichuan,Aba	Jin,L et al.2013
AT045	JX068380	Aba Tibetan pig	Sichuan,Aba	Jin,L et al.2013
AT046	JX068379	Aba Tibetan pig	Sichuan,Aba	Jin,L et al.2013
AT047	JX068378	Aba Tibetan pig	Sichuan,Aba	Jin,L et al.2013
AT048	JX068377	Aba Tibetan pig	Sichuan,Aba	Jin,L et al.2013
AT049	JX068376	Aba Tibetan pig	Sichuan,Aba	Jin,L et al.2013
AT050	JX068375	Aba Tibetan pig	Sichuan,Aba	Jin,L et al.2013
AT051	JX068374	Aba Tibetan pig	Sichuan,Aba	Jin,L et al.2013
AT052	JX068373	Aba Tibetan pig	Sichuan,Aba	Jin,L et al.2013
AT053	JX068372	Aba Tibetan pig	Sichuan,Aba	Jin,L et al.2013
AT054	JX068371	Aba Tibetan pig	Sichuan,Aba	Jin,L et al.2013
AT055	JX068370	Aba Tibetan pig	Sichuan,Aba	Jin,L et al.2013
AT056	JX068369	Aba Tibetan pig	Sichuan,Aba	Jin,L et al.2013
AT057	JX068368	Aba Tibetan pig	Sichuan,Aba	Jin,L et al.2013
AT058	JX068367	Aba Tibetan pig	Sichuan,Aba	Jin,L et al.2013
AT059	JX068366	Aba Tibetan pig	Sichuan,Aba	Jin,L et al.2013
AT060	JX068365	Aba Tibetan pig	Sichuan,Aba	Jin,L et al.2013
AT061	JX068364	Aba Tibetan pig	Sichuan,Aba	Jin,L et al.2013
AT062	JX068363	Aba Tibetan pig	Sichuan,Aba	Jin,L et al.2013
AT063	JX068362	Aba Tibetan pig	Sichuan,Aba	Jin,L et al.2013
AT064	JX068361	Aba Tibetan pig	Sichuan,Aba	Jin,L et al.2013
AT065	JX068360	Aba Tibetan pig	Sichuan,Aba	Jin,L et al.2013
AT066	JX068359	Aba Tibetan pig	Sichuan,Aba	Jin,L et al.2013
AT067	JX068358	Aba Tibetan pig	Sichuan,Aba	Jin,L et al.2013
AT068	JX068357	Aba Tibetan pig	Sichuan,Aba	Jin,L et al.2013
AT069	JX068356	Aba Tibetan pig	Sichuan,Aba	Jin,L et al.2013
AT070	JX068355	Aba Tibetan pig	Sichuan,Aba	Jin,L et al.2013
GT001	JX068306	Ganzi Tibetan pig	Sichuan,Ganzi	Jin,L et al.2013
GT002	JX068305	Ganzi Tibetan pig	Sichuan,Ganzi	Jin,L et al.2013
GT003	JX068304	Ganzi Tibetan pig	Sichuan,Ganzi	Jin,L et al.2013
GT004	JX068303	Ganzi Tibetan pig	Sichuan,Ganzi	Jin,L et al.2013
GT005	JX068302	Ganzi Tibetan pig	Sichuan,Ganzi	Jin,L et al.2013
GT006	JX068301	Ganzi Tibetan pig	Sichuan,Ganzi	Jin,L et al.2013
GT007	JX068300	Ganzi Tibetan pig	Sichuan,Ganzi	Jin,L et al.2013
GT008	JX068299	Ganzi Tibetan pig	Sichuan,Ganzi	Jin,L et al.2013
GT009	JX068298	Ganzi Tibetan pig	Sichuan,Ganzi	Jin,L et al.2013
GT010	JX068297	Ganzi Tibetan pig	Sichuan,Ganzi	Jin,L et al.2013
GT011	JX068296	Ganzi Tibetan pig	Sichuan,Ganzi	Jin,L et al.2013
GT012	JX068295	Ganzi Tibetan pig	Sichuan,Ganzi	Jin,L et al.2013
GT013	JX068294	Ganzi Tibetan pig	Sichuan,Ganzi	Jin,L et al.2013
GT014	JX068293	Ganzi Tibetan pig	Sichuan,Ganzi	Jin,L et al.2013
GT015	JX068292	Ganzi Tibetan pig	Sichuan,Ganzi	Jin,L et al.2013
GT016	JX068291	Ganzi Tibetan pig	Sichuan,Ganzi	Jin,L et al.2013
GT017	JX068290	Ganzi Tibetan pig	Sichuan,Ganzi	Jin,L et al.2013
GT018	JX068289	Ganzi Tibetan pig	Sichuan,Ganzi	Jin,L et al.2013
GT019	JX068288	Ganzi Tibetan pig	Sichuan,Ganzi	Jin,L et al.2013
GT020	JX068287	Ganzi Tibetan pig	Sichuan,Ganzi	Jin,L et al.2013
GT021	JX068286	Ganzi Tibetan pig	Sichuan,Ganzi	Jin,L et al.2013
GT022	JX068285	Ganzi Tibetan pig	Sichuan,Ganzi	Jin,L et al.2013
GT023	JX068284	Ganzi Tibetan pig	Sichuan,Ganzi	Jin,L et al.2013
GT024	JX068283	Ganzi Tibetan pig	Sichuan,Ganzi	Jin,L et al.2013
GT025	JX068282	Ganzi Tibetan pig	Sichuan,Ganzi	Jin,L et al.2013
GT026	JX068281	Ganzi Tibetan pig	Sichuan,Ganzi	Jin,L et al.2013
GT027	JX068280	Ganzi Tibetan pig	Sichuan,Ganzi	Jin,L et al.2013
GT028	JX068279	Ganzi Tibetan pig	Sichuan,Ganzi	Jin,L et al.2013
GT029	JX068278	Ganzi Tibetan pig	Sichuan,Ganzi	Jin,L et al.2013
GT030	JX068277	Ganzi Tibetan pig	Sichuan,Ganzi	Jin,L et al.2013
GT031	JX068276	Ganzi Tibetan pig	Sichuan,Ganzi	Jin,L et al.2013
GT032	JX068275	Ganzi Tibetan pig	Sichuan,Ganzi	Jin,L et al.2013
GT033	HQ148325	Ganzi Tibetan pig	Sichuan,Ganzi	Shuli Yang et al.2011
GT034	HQ148324	Ganzi Tibetan pig	Sichuan,Ganzi	Shuli Yang et al.2011
GT035	HQ148323	Ganzi Tibetan pig	Sichuan,Ganzi	Shuli Yang et al.2011
GT036	HQ148322	Ganzi Tibetan pig	Sichuan,Ganzi	Shuli Yang et al.2011
GT037	HQ148321	Ganzi Tibetan pig	Sichuan,Ganzi	Shuli Yang et al.2011
GT038	HQ148320	Ganzi Tibetan pig	Sichuan,Ganzi	Shuli Yang et al.2011
GT039	HQ148319	Ganzi Tibetan pig	Sichuan,Ganzi	Shuli Yang et al.2011
GT040	HQ148318	Ganzi Tibetan pig	Sichuan,Ganzi	Shuli Yang et al.2011
GT041	HQ148317	Ganzi Tibetan pig	Sichuan,Ganzi	Shuli Yang et al.2011
GT042	HQ148316	Ganzi Tibetan pig	Sichuan,Ganzi	Shuli Yang et al.2011
GT043	DQ496435	Ganzi Tibetan pig	Sichuan,Ganzi	Gui-Sheng Wu et al.2007
GT044	DQ496434	Ganzi Tibetan pig	Sichuan,Ganzi	Gui-Sheng Wu et al.2007
GT045	DQ496432	Ganzi Tibetan pig	Sichuan,Ganzi	Gui-Sheng Wu et al.2007
GT046	DQ496431	Ganzi Tibetan pig	Sichuan,Ganzi	Gui-Sheng Wu et al.2007
GT047	DQ496430	Ganzi Tibetan pig	Sichuan,Ganzi	Gui-Sheng Wu et al.2007
GT048	DQ496429	Ganzi Tibetan pig	Sichuan,Ganzi	Gui-Sheng Wu et al.2007
GT049	DQ496427	Ganzi Tibetan pig	Sichuan,Ganzi	Gui-Sheng Wu et al.2007
GT050	DQ496426	Ganzi Tibetan pig	Sichuan,Ganzi	Gui-Sheng Wu et al.2007
GT051	DQ496425	Ganzi Tibetan pig	Sichuan,Ganzi	Gui-Sheng Wu et al.2007
GT052	DQ496424	Ganzi Tibetan pig	Sichuan,Ganzi	Gui-Sheng Wu et al.2007
GT053	DQ496433	Ganzi Tibetan pig	Sichuan,Ganzi	Gui-Sheng Wu et al.2007
GT054	DQ496428	Ganzi Tibetan pig	Sichuan,Ganzi	Gui-Sheng Wu et al.2007
GT055		Ganzi Tibetan pig	Sichuan,Ganzi	This study
GT056		Ganzi Tibetan pig	Sichuan,Ganzi	This study
GT057		Ganzi Tibetan pig	Sichuan,Ganzi	This study
GT058		Ganzi Tibetan pig	Sichuan,Ganzi	This study
GT059		Ganzi Tibetan pig	Sichuan,Ganzi	This study
GT060		Ganzi Tibetan pig	Sichuan,Ganzi	This study
GT061		Ganzi Tibetan pig	Sichuan,Ganzi	This study
GT062		Ganzi Tibetan pig	Sichuan,Ganzi	This study
GT063		Ganzi Tibetan pig	Sichuan,Ganzi	This study
GT064		Ganzi Tibetan pig	Sichuan,Ganzi	This study
GT065		Ganzi Tibetan pig	Sichuan,Ganzi	This study
GT066		Ganzi Tibetan pig	Sichuan,Ganzi	This study
GT067		Ganzi Tibetan pig	Sichuan,Ganzi	This study
GT068		Ganzi Tibetan pig	Sichuan,Ganzi	This study
GT069		Ganzi Tibetan pig	Sichuan,Ganzi	This study
GT070		Ganzi Tibetan pig	Sichuan,Ganzi	This study
GT071		Ganzi Tibetan pig	Sichuan,Ganzi	This study
GT072		Ganzi Tibetan pig	Sichuan,Ganzi	This study
GT073		Ganzi Tibetan pig	Sichuan,Ganzi	This study
GT074		Ganzi Tibetan pig	Sichuan,Ganzi	This study
GT075		Ganzi Tibetan pig	Sichuan,Ganzi	This study
GT076		Ganzi Tibetan pig	Sichuan,Ganzi	This study
GT077		Ganzi Tibetan pig	Sichuan,Ganzi	This study
GT078		Ganzi Tibetan pig	Sichuan,Ganzi	This study
GT079		Ganzi Tibetan pig	Sichuan,Ganzi	This study
GT080		Ganzi Tibetan pig	Sichuan,Ganzi	This study
GT081		Ganzi Tibetan pig	Sichuan,Ganzi	This study
GT082		Ganzi Tibetan pig	Sichuan,Ganzi	This study
GT083		Ganzi Tibetan pig	Sichuan,Ganzi	This study
GT084		Ganzi Tibetan pig	Sichuan,Ganzi	This study
GT085		Ganzi Tibetan pig	Sichuan,Ganzi	This study
GT086		Ganzi Tibetan pig	Sichuan,Ganzi	This study
GT087		Ganzi Tibetan pig	Sichuan,Ganzi	This study
GT088		Ganzi Tibetan pig	Sichuan,Ganzi	This study
GT089		Ganzi Tibetan pig	Sichuan,Ganzi	This study
GT090		Ganzi Tibetan pig	Sichuan,Ganzi	This study
GT091		Ganzi Tibetan pig	Sichuan,Ganzi	This study
GT092		Ganzi Tibetan pig	Sichuan,Ganzi	This study
GT093		Ganzi Tibetan pig	Sichuan,Ganzi	This study
GT094		Ganzi Tibetan pig	Sichuan,Ganzi	This study
GT095		Ganzi Tibetan pig	Sichuan,Ganzi	This study
GT096		Ganzi Tibetan pig	Sichuan,Ganzi	This study
GT097		Ganzi Tibetan pig	Sichuan,Ganzi	This study
GT098		Ganzi Tibetan pig	Sichuan,Ganzi	This study
GT099		Ganzi Tibetan pig	Sichuan,Ganzi	This study
GT100		Ganzi Tibetan pig	Sichuan,Ganzi	This study
GT101		Ganzi Tibetan pig	Sichuan,Ganzi	This study
GT102		Ganzi Tibetan pig	Sichuan,Ganzi	This study
GT103		Ganzi Tibetan pig	Sichuan,Ganzi	This study
GT104		Ganzi Tibetan pig	Sichuan,Ganzi	This study
GT105		Ganzi Tibetan pig	Sichuan,Ganzi	This study
GT106		Ganzi Tibetan pig	Sichuan,Ganzi	This study
GT107		Ganzi Tibetan pig	Sichuan,Ganzi	This study
GT108		Ganzi Tibetan pig	Sichuan,Ganzi	This study
GT109		Ganzi Tibetan pig	Sichuan,Ganzi	This study
GT110		Ganzi Tibetan pig	Sichuan,Ganzi	This study
GT111		Ganzi Tibetan pig	Sichuan,Ganzi	This study
GT112		Ganzi Tibetan pig	Sichuan,Ganzi	This study
GT113		Ganzi Tibetan pig	Sichuan,Ganzi	This study
GT114		Ganzi Tibetan pig	Sichuan,Ganzi	This study
GT115		Ganzi Tibetan pig	Sichuan,Ganzi	This study
GT116		Ganzi Tibetan pig	Sichuan,Ganzi	This study
GT117		Ganzi Tibetan pig	Sichuan,Ganzi	This study
GT118		Ganzi Tibetan pig	Sichuan,Ganzi	This study
GT119		Ganzi Tibetan pig	Sichuan,Ganzi	This study
GT120		Ganzi Tibetan pig	Sichuan,Ganzi	This study
GT121		Ganzi Tibetan pig	Sichuan,Ganzi	This study
GT122		Ganzi Tibetan pig	Sichuan,Ganzi	This study
GT123		Ganzi Tibetan pig	Sichuan,Ganzi	This study
GT124		Ganzi Tibetan pig	Sichuan,Ganzi	This study
GT125		Ganzi Tibetan pig	Sichuan,Ganzi	This study
GT126		Ganzi Tibetan pig	Sichuan,Ganzi	This study
GT127		Ganzi Tibetan pig	Sichuan,Ganzi	This study
GT128		Ganzi Tibetan pig	Sichuan,Ganzi	This study
GT129		Ganzi Tibetan pig	Sichuan,Ganzi	This study
GT130		Ganzi Tibetan pig	Sichuan,Ganzi	This study
GT131		Ganzi Tibetan pig	Sichuan,Ganzi	This study
GT132		Ganzi Tibetan pig	Sichuan,Ganzi	This study
GT133		Ganzi Tibetan pig	Sichuan,Ganzi	This study
DT001	DQ496409	Diqing Tibetan pig	Yunnan,Diqing	Gui-Sheng Wu et al.2007
DT002	DQ496408	Diqing Tibetan pig	Yunnan,Diqing	Gui-Sheng Wu et al.2007
DT003	DQ496407	Diqing Tibetan pig	Yunnan,Diqing	Gui-Sheng Wu et al.2007
DT004	DQ496406	Diqing Tibetan pig	Yunnan,Diqing	Gui-Sheng Wu et al.2007
DT005	DQ496405	Diqing Tibetan pig	Yunnan,Diqing	Gui-Sheng Wu et al.2007
DT006	DQ496404	Diqing Tibetan pig	Yunnan,Diqing	Gui-Sheng Wu et al.2007
DT007	DQ496403	Diqing Tibetan pig	Yunnan,Diqing	Gui-Sheng Wu et al.2007
DT008	DQ496402	Diqing Tibetan pig	Yunnan,Diqing	Gui-Sheng Wu et al.2007
DT009	DQ496401	Diqing Tibetan pig	Yunnan,Diqing	Gui-Sheng Wu et al.2007
DT010	DQ496400	Diqing Tibetan pig	Yunnan,Diqing	Gui-Sheng Wu et al.2007
DT011	DQ496399	Diqing Tibetan pig	Yunnan,Diqing	Gui-Sheng Wu et al.2007
DT012	DQ496398	Diqing Tibetan pig	Yunnan,Diqing	Gui-Sheng Wu et al.2007
DT013	DQ496397	Diqing Tibetan pig	Yunnan,Diqing	Gui-Sheng Wu et al.2007
DT014	DQ496396	Diqing Tibetan pig	Yunnan,Diqing	Gui-Sheng Wu et al.2007
DT015	DQ496395	Diqing Tibetan pig	Yunnan,Diqing	Gui-Sheng Wu et al.2007
DT016	DQ496394	Diqing Tibetan pig	Yunnan,Diqing	Gui-Sheng Wu et al.2007
DT017	DQ496392	Diqing Tibetan pig	Yunnan,Diqing	Gui-Sheng Wu et al.2007
DT018	EF545576	Diqing Tibetan pig	Yunnan,Diqing	Gui-Sheng Wu et al.2007
DT019	HQ148449	Diqing Tibetan pig	Yunnan,Diqing	Shuli Yang et al.2011
DT020	HQ148448	Diqing Tibetan pig	Yunnan,Diqing	Shuli Yang et al.2011
DT021	HQ148447	Diqing Tibetan pig	Yunnan,Diqing	Shuli Yang et al.2011
DT022	HQ148446	Diqing Tibetan pig	Yunnan,Diqing	Shuli Yang et al.2011
DT023	HQ148445	Diqing Tibetan pig	Yunnan,Diqing	Shuli Yang et al.2011
DT024	HQ148444	Diqing Tibetan pig	Yunnan,Diqing	Shuli Yang et al.2011
DT025	HQ148443	Diqing Tibetan pig	Yunnan,Diqing	Shuli Yang et al.2011
DT026	HQ148442	Diqing Tibetan pig	Yunnan,Diqing	Shuli Yang et al.2011
DT027	HQ148441	Diqing Tibetan pig	Yunnan,Diqing	Shuli Yang et al.2011
DT028	HQ148440	Diqing Tibetan pig	Yunnan,Diqing	Shuli Yang et al.2011
DT029	HQ148439	Diqing Tibetan pig	Yunnan,Diqing	Shuli Yang et al.2011
DT030	HQ148438	Diqing Tibetan pig	Yunnan,Diqing	Shuli Yang et al.2011
DT031	HQ148437	Diqing Tibetan pig	Yunnan,Diqing	Shuli Yang et al.2011
DT032	HQ148436	Diqing Tibetan pig	Yunnan,Diqing	Shuli Yang et al.2011
DT033	HQ148435	Diqing Tibetan pig	Yunnan,Diqing	Shuli Yang et al.2011
DT034	HQ148434	Diqing Tibetan pig	Yunnan,Diqing	Shuli Yang et al.2011
DT035	HQ148433	Diqing Tibetan pig	Yunnan,Diqing	Shuli Yang et al.2011
DT036	HQ148432	Diqing Tibetan pig	Yunnan,Diqing	Shuli Yang et al.2011
DT037	HQ148431	Diqing Tibetan pig	Yunnan,Diqing	Shuli Yang et al.2011
DT038	HQ148430	Diqing Tibetan pig	Yunnan,Diqing	Shuli Yang et al.2011
DT039	HQ148429	Diqing Tibetan pig	Yunnan,Diqing	Shuli Yang et al.2011
DT040	HQ148428	Diqing Tibetan pig	Yunnan,Diqing	Shuli Yang et al.2011
DT041	HQ148427	Diqing Tibetan pig	Yunnan,Diqing	Shuli Yang et al.2011
DT042	HQ148426	Diqing Tibetan pig	Yunnan,Diqing	Shuli Yang et al.2011
DT043	HQ148425	Diqing Tibetan pig	Yunnan,Diqing	Shuli Yang et al.2011
DT044	HQ148424	Diqing Tibetan pig	Yunnan,Diqing	Shuli Yang et al.2011
DT045	HQ148423	Diqing Tibetan pig	Yunnan,Diqing	Shuli Yang et al.2011
DT046	HQ148422	Diqing Tibetan pig	Yunnan,Diqing	Shuli Yang et al.2011
DT047	HQ148421	Diqing Tibetan pig	Yunnan,Diqing	Shuli Yang et al.2011
DT048	HQ148420	Diqing Tibetan pig	Yunnan,Diqing	Shuli Yang et al.2011
DT049	HQ148419	Diqing Tibetan pig	Yunnan,Diqing	Shuli Yang et al.2011
DT050	HQ148418	Diqing Tibetan pig	Yunnan,Diqing	Shuli Yang et al.2011
DT051	HQ148417	Diqing Tibetan pig	Yunnan,Diqing	Shuli Yang et al.2011
DT052	HQ148416	Diqing Tibetan pig	Yunnan,Diqing	Shuli Yang et al.2011
DT053	HQ148415	Diqing Tibetan pig	Yunnan,Diqing	Shuli Yang et al.2011
DT054	HQ148414	Diqing Tibetan pig	Yunnan,Diqing	Shuli Yang et al.2011
DT055	HQ148413	Diqing Tibetan pig	Yunnan,Diqing	Shuli Yang et al.2011
DT056	HQ148412	Diqing Tibetan pig	Yunnan,Diqing	Shuli Yang et al.2011
DT057	HQ148411	Diqing Tibetan pig	Yunnan,Diqing	Shuli Yang et al.2011
DT058	JX068274	Diqing Tibetan pig	Yunnan,Diqing	Jin,L et al.2013
DT059	JX068273	Diqing Tibetan pig	Yunnan,Diqing	Jin,L et al.2013
DT060	JX068272	Diqing Tibetan pig	Yunnan,Diqing	Jin,L et al.2013
DT061	JX068271	Diqing Tibetan pig	Yunnan,Diqing	Jin,L et al.2013
DT062	JX068270	Diqing Tibetan pig	Yunnan,Diqing	Jin,L et al.2013
DT063	JX068269	Diqing Tibetan pig	Yunnan,Diqing	Jin,L et al.2013
DT064	JX068268	Diqing Tibetan pig	Yunnan,Diqing	Jin,L et al.2013
DT065	JX068267	Diqing Tibetan pig	Yunnan,Diqing	Jin,L et al.2013
DT066	JX068266	Diqing Tibetan pig	Yunnan,Diqing	Jin,L et al.2013
DT067	JX068265	Diqing Tibetan pig	Yunnan,Diqing	Jin,L et al.2013
DT068	JX068264	Diqing Tibetan pig	Yunnan,Diqing	Jin,L et al.2013
DT069	JX068263	Diqing Tibetan pig	Yunnan,Diqing	Jin,L et al.2013
DT070	JX068262	Diqing Tibetan pig	Yunnan,Diqing	Jin,L et al.2013
DT071	JX068261	Diqing Tibetan pig	Yunnan,Diqing	Jin,L et al.2013
DT072	JX068260	Diqing Tibetan pig	Yunnan,Diqing	Jin,L et al.2013
DT073	JX068259	Diqing Tibetan pig	Yunnan,Diqing	Jin,L et al.2013
DT074	JX068258	Diqing Tibetan pig	Yunnan,Diqing	Jin,L et al.2013
DT075	JX068257	Diqing Tibetan pig	Yunnan,Diqing	Jin,L et al.2013
DT076	JX068256	Diqing Tibetan pig	Yunnan,Diqing	Jin,L et al.2013
DT077	JX068255	Diqing Tibetan pig	Yunnan,Diqing	Jin,L et al.2013
DT078	JX068254	Diqing Tibetan pig	Yunnan,Diqing	Jin,L et al.2013
DT079	JX068253	Diqing Tibetan pig	Yunnan,Diqing	Jin,L et al.2013
DT080	JX068252	Diqing Tibetan pig	Yunnan,Diqing	Jin,L et al.2013
DT081	JX068251	Diqing Tibetan pig	Yunnan,Diqing	Jin,L et al.2013
DT082	JX068250	Diqing Tibetan pig	Yunnan,Diqing	Jin,L et al.2013
DT083	JX068249	Diqing Tibetan pig	Yunnan,Diqing	Jin,L et al.2013
DT084	JX068248	Diqing Tibetan pig	Yunnan,Diqing	Jin,L et al.2013
DT085	JX068247	Diqing Tibetan pig	Yunnan,Diqing	Jin,L et al.2013
DT086	JX068246	Diqing Tibetan pig	Yunnan,Diqing	Jin,L et al.2013
DT087	JX068245	Diqing Tibetan pig	Yunnan,Diqing	Jin,L et al.2013
DT088	JX068244	Diqing Tibetan pig	Yunnan,Diqing	Jin,L et al.2013
DT089	JX068243	Diqing Tibetan pig	Yunnan,Diqing	Jin,L et al.2013
DT090	JX068242	Diqing Tibetan pig	Yunnan,Diqing	Jin,L et al.2013
DT091	JX068241	Diqing Tibetan pig	Yunnan,Diqing	Jin,L et al.2013
DT092	JX068240	Diqing Tibetan pig	Yunnan,Diqing	Jin,L et al.2013
DT093	JX068239	Diqing Tibetan pig	Yunnan,Diqing	Jin,L et al.2013
DT094	JX068238	Diqing Tibetan pig	Yunnan,Diqing	Jin,L et al.2013
DT095	JX068237	Diqing Tibetan pig	Yunnan,Diqing	Jin,L et al.2013
DT096		Diqing Tibetan pig	Yunnan,Diqing	This study
DT097		Diqing Tibetan pig	Yunnan,Diqing	This study
DT098		Diqing Tibetan pig	Yunnan,Diqing	This study
DT099		Diqing Tibetan pig	Yunnan,Diqing	This study
DT100		Diqing Tibetan pig	Yunnan,Diqing	This study
DT101		Diqing Tibetan pig	Yunnan,Diqing	This study
DT102		Diqing Tibetan pig	Yunnan,Diqing	This study
DT103		Diqing Tibetan pig	Yunnan,Diqing	This study
DT104		Diqing Tibetan pig	Yunnan,Diqing	This study
DT105		Diqing Tibetan pig	Yunnan,Diqing	This study
DT106		Diqing Tibetan pig	Yunnan,Diqing	This study
DT107		Diqing Tibetan pig	Yunnan,Diqing	This study
DT108		Diqing Tibetan pig	Yunnan,Diqing	This study
DT109		Diqing Tibetan pig	Yunnan,Diqing	This study
DT110		Diqing Tibetan pig	Yunnan,Diqing	This study
DT111		Diqing Tibetan pig	Yunnan,Diqing	This study
DT112		Diqing Tibetan pig	Yunnan,Diqing	This study
DT113		Diqing Tibetan pig	Yunnan,Diqing	This study
DT114		Diqing Tibetan pig	Yunnan,Diqing	This study
DT115		Diqing Tibetan pig	Yunnan,Diqing	This study
DT116		Diqing Tibetan pig	Yunnan,Diqing	This study
DT117		Diqing Tibetan pig	Yunnan,Diqing	This study
DT118		Diqing Tibetan pig	Yunnan,Diqing	This study
DT119		Diqing Tibetan pig	Yunnan,Diqing	This study
DT120		Diqing Tibetan pig	Yunnan,Diqing	This study
DT121		Diqing Tibetan pig	Yunnan,Diqing	This study
DT122		Diqing Tibetan pig	Yunnan,Diqing	This study
DT123		Diqing Tibetan pig	Yunnan,Diqing	This study
DT124		Diqing Tibetan pig	Yunnan,Diqing	This study
DT125		Diqing Tibetan pig	Yunnan,Diqing	This study
DT126		Diqing Tibetan pig	Yunnan,Diqing	This study
DT127		Diqing Tibetan pig	Yunnan,Diqing	This study
DT128		Diqing Tibetan pig	Yunnan,Diqing	This study
DT129		Diqing Tibetan pig	Yunnan,Diqing	This study
DT130		Diqing Tibetan pig	Yunnan,Diqing	This study
DT131		Diqing Tibetan pig	Yunnan,Diqing	This study
DT132		Diqing Tibetan pig	Yunnan,Diqing	This study
DT133		Diqing Tibetan pig	Yunnan,Diqing	This study
DT134		Diqing Tibetan pig	Yunnan,Diqing	This study
DT135		Diqing Tibetan pig	Yunnan,Diqing	This study
DT136		Diqing Tibetan pig	Yunnan,Diqing	This study
DT137		Diqing Tibetan pig	Yunnan,Diqing	This study
DT138		Diqing Tibetan pig	Yunnan,Diqing	This study
DT139		Diqing Tibetan pig	Yunnan,Diqing	This study
DT140		Diqing Tibetan pig	Yunnan,Diqing	This study
DT141		Diqing Tibetan pig	Yunnan,Diqing	This study
DT142		Diqing Tibetan pig	Yunnan,Diqing	This study
DT143		Diqing Tibetan pig	Yunnan,Diqing	This study
DT144		Diqing Tibetan pig	Yunnan,Diqing	This study
DT145		Diqing Tibetan pig	Yunnan,Diqing	This study
DT146		Diqing Tibetan pig	Yunnan,Diqing	This study
DT147		Diqing Tibetan pig	Yunnan,Diqing	This study
DT148		Diqing Tibetan pig	Yunnan,Diqing	This study
DT149		Diqing Tibetan pig	Yunnan,Diqing	This study
DT150		Diqing Tibetan pig	Yunnan,Diqing	This study
DT151		Diqing Tibetan pig	Yunnan,Diqing	This study
DT152		Diqing Tibetan pig	Yunnan,Diqing	This study
DT153		Diqing Tibetan pig	Yunnan,Diqing	This study
DT154		Diqing Tibetan pig	Yunnan,Diqing	This study
DT155		Diqing Tibetan pig	Yunnan,Diqing	This study
DT156		Diqing Tibetan pig	Yunnan,Diqing	This study
DT157		Diqing Tibetan pig	Yunnan,Diqing	This study
DT158		Diqing Tibetan pig	Yunnan,Diqing	This study
DT159		Diqing Tibetan pig	Yunnan,Diqing	This study
DT160		Diqing Tibetan pig	Yunnan,Diqing	This study
DT161		Diqing Tibetan pig	Yunnan,Diqing	This study
DT162		Diqing Tibetan pig	Yunnan,Diqing	This study
DT163		Diqing Tibetan pig	Yunnan,Diqing	This study
DT164		Diqing Tibetan pig	Yunnan,Diqing	This study
DT165		Diqing Tibetan pig	Yunnan,Diqing	This study
DT166		Diqing Tibetan pig	Yunnan,Diqing	This study
DT167		Diqing Tibetan pig	Yunnan,Diqing	This study
DT168		Diqing Tibetan pig	Yunnan,Diqing	This study
DT169		Diqing Tibetan pig	Yunnan,Diqing	This study
DT170		Diqing Tibetan pig	Yunnan,Diqing	This study
DT171		Diqing Tibetan pig	Yunnan,Diqing	This study
DT172		Diqing Tibetan pig	Yunnan,Diqing	This study
DT173		Diqing Tibetan pig	Yunnan,Diqing	This study
DT174		Diqing Tibetan pig	Yunnan,Diqing	This study
DT175		Diqing Tibetan pig	Yunnan,Diqing	This study
DT176		Diqing Tibetan pig	Yunnan,Diqing	This study
DT177		Diqing Tibetan pig	Yunnan,Diqing	This study
DT178		Diqing Tibetan pig	Yunnan,Diqing	This study
LT001	JX068236	Linzhi Tibetan pig	Tibet,Linzhi	Jin,L et al.2013
LT002	JX068235	Linzhi Tibetan pig	Tibet,Linzhi	Jin,L et al.2013
LT003	JX068234	Linzhi Tibetan pig	Tibet,Linzhi	Jin,L et al.2013
LT004	JX068233	Linzhi Tibetan pig	Tibet,Linzhi	Jin,L et al.2013
LT005	JX068232	Linzhi Tibetan pig	Tibet,Linzhi	Jin,L et al.2013
LT006	JX068231	Linzhi Tibetan pig	Tibet,Linzhi	Jin,L et al.2013
LT007	JX068230	Linzhi Tibetan pig	Tibet,Linzhi	Jin,L et al.2013
LT008	JX068229	Linzhi Tibetan pig	Tibet,Linzhi	Jin,L et al.2013
LT009	JX068228	Linzhi Tibetan pig	Tibet,Linzhi	Jin,L et al.2013
LT010	JX068227	Linzhi Tibetan pig	Tibet,Linzhi	Jin,L et al.2013
LT011	JX068226	Linzhi Tibetan pig	Tibet,Linzhi	Jin,L et al.2013
LT012	JX068225	Linzhi Tibetan pig	Tibet,Linzhi	Jin,L et al.2013
LT013	JX068224	Linzhi Tibetan pig	Tibet,Linzhi	Jin,L et al.2013
LT014	JX068223	Linzhi Tibetan pig	Tibet,Linzhi	Jin,L et al.2013
LT015	JX068222	Linzhi Tibetan pig	Tibet,Linzhi	Jin,L et al.2013
LT016	JX068221	Linzhi Tibetan pig	Tibet,Linzhi	Jin,L et al.2013
LT017	JX068220	Linzhi Tibetan pig	Tibet,Linzhi	Jin,L et al.2013
LT018	JX068219	Linzhi Tibetan pig	Tibet,Linzhi	Jin,L et al.2013
LT019	JX068218	Linzhi Tibetan pig	Tibet,Linzhi	Jin,L et al.2013
LT020	JX068217	Linzhi Tibetan pig	Tibet,Linzhi	Jin,L et al.2013
LT021	JX068216	Linzhi Tibetan pig	Tibet,Linzhi	Jin,L et al.2013
LT022	JX068215	Linzhi Tibetan pig	Tibet,Linzhi	Jin,L et al.2013
LT023	JX068214	Linzhi Tibetan pig	Tibet,Linzhi	Jin,L et al.2013
LT024	JX068213	Linzhi Tibetan pig	Tibet,Linzhi	Jin,L et al.2013
LT025	JX068212	Linzhi Tibetan pig	Tibet,Linzhi	Jin,L et al.2013
LT026	JX068211	Linzhi Tibetan pig	Tibet,Linzhi	Jin,L et al.2013
LT027	JX068210	Linzhi Tibetan pig	Tibet,Linzhi	Jin,L et al.2013
LT028	JX068209	Linzhi Tibetan pig	Tibet,Linzhi	Jin,L et al.2013
LT029	JX068208	Linzhi Tibetan pig	Tibet,Linzhi	Jin,L et al.2013
LT030	JX068207	Linzhi Tibetan pig	Tibet,Linzhi	Jin,L et al.2013
LT031	JX068206	Linzhi Tibetan pig	Tibet,Linzhi	Jin,L et al.2013
LT032	JX068205	Linzhi Tibetan pig	Tibet,Linzhi	Jin,L et al.2013
LT033	JX068204	Linzhi Tibetan pig	Tibet,Linzhi	Jin,L et al.2013
LT034	JX068203	Linzhi Tibetan pig	Tibet,Linzhi	Jin,L et al.2013
LT035	JX068202	Linzhi Tibetan pig	Tibet,Linzhi	Jin,L et al.2013
LT036	JX068201	Linzhi Tibetan pig	Tibet,Linzhi	Jin,L et al.2013
LT037	JX068200	Linzhi Tibetan pig	Tibet,Linzhi	Jin,L et al.2013
LT038	JX068199	Linzhi Tibetan pig	Tibet,Linzhi	Jin,L et al.2013
LT039	JX068198	Linzhi Tibetan pig	Tibet,Linzhi	Jin,L et al.2013
LT040	JX068197	Linzhi Tibetan pig	Tibet,Linzhi	Jin,L et al.2013
LT041	JX068196	Linzhi Tibetan pig	Tibet,Linzhi	Jin,L et al.2013
LT042	JX068195	Linzhi Tibetan pig	Tibet,Linzhi	Jin,L et al.2013
LT043	JX068194	Linzhi Tibetan pig	Tibet,Linzhi	Jin,L et al.2013
LT044	JX068193	Linzhi Tibetan pig	Tibet,Linzhi	Jin,L et al.2013
LT045	JX068192	Linzhi Tibetan pig	Tibet,Linzhi	Jin,L et al.2013
LT046	JX068191	Linzhi Tibetan pig	Tibet,Linzhi	Jin,L et al.2013
LT047	JX068190	Linzhi Tibetan pig	Tibet,Linzhi	Jin,L et al.2013
LT048	JX068189	Linzhi Tibetan pig	Tibet,Linzhi	Jin,L et al.2013
LT049	JX068188	Linzhi Tibetan pig	Tibet,Linzhi	Jin,L et al.2013
LT050	JX068187	Linzhi Tibetan pig	Tibet,Linzhi	Jin,L et al.2013
LT051	JX068186	Linzhi Tibetan pig	Tibet,Linzhi	Jin,L et al.2013
LT052	JX068185	Linzhi Tibetan pig	Tibet,Linzhi	Jin,L et al.2013
LT053	JX068184	Linzhi Tibetan pig	Tibet,Linzhi	Jin,L et al.2013
LT054	JX068183	Linzhi Tibetan pig	Tibet,Linzhi	Jin,L et al.2013
LT055	JX068182	Linzhi Tibetan pig	Tibet,Linzhi	Jin,L et al.2013
LT056	JX068181	Linzhi Tibetan pig	Tibet,Linzhi	Jin,L et al.2013
LT057	JX068180	Linzhi Tibetan pig	Tibet,Linzhi	Jin,L et al.2013
LT058	JX068179	Linzhi Tibetan pig	Tibet,Linzhi	Jin,L et al.2013
LT059	JX068178	Linzhi Tibetan pig	Tibet,Linzhi	Jin,L et al.2013
LT060	JX068177	Linzhi Tibetan pig	Tibet,Linzhi	Jin,L et al.2013
LT061	JX068176	Linzhi Tibetan pig	Tibet,Linzhi	Jin,L et al.2013
LT062	JX068175	Linzhi Tibetan pig	Tibet,Linzhi	Jin,L et al.2013
LT063	JX068174	Linzhi Tibetan pig	Tibet,Linzhi	Jin,L et al.2013
LT064	JX068173	Linzhi Tibetan pig	Tibet,Linzhi	Jin,L et al.2013
LT065	JX068172	Linzhi Tibetan pig	Tibet,Linzhi	Jin,L et al.2013
LT066	JX068171	Linzhi Tibetan pig	Tibet,Linzhi	Jin,L et al.2013
LT067	JX068170	Linzhi Tibetan pig	Tibet,Linzhi	Jin,L et al.2013
LT068	JX068169	Linzhi Tibetan pig	Tibet,Linzhi	Jin,L et al.2013
LT069	JX068168	Linzhi Tibetan pig	Tibet,Linzhi	Jin,L et al.2013
LT070	JX068167	Linzhi Tibetan pig	Tibet,Linzhi	Jin,L et al.2013
LT071	JX068166	Linzhi Tibetan pig	Tibet,Linzhi	Jin,L et al.2013
LT072	JX068165	Linzhi Tibetan pig	Tibet,Linzhi	Jin,L et al.2013
LT073	JX068164	Linzhi Tibetan pig	Tibet,Linzhi	Jin,L et al.2013
LT074	JX068163	Linzhi Tibetan pig	Tibet,Linzhi	Jin,L et al.2013
LT075	JX068162	Linzhi Tibetan pig	Tibet,Linzhi	Jin,L et al.2013
LT076	JX068161	Linzhi Tibetan pig	Tibet,Linzhi	Jin,L et al.2013
LT077	JX068160	Linzhi Tibetan pig	Tibet,Linzhi	Jin,L et al.2013
LT078	JX068159	Linzhi Tibetan pig	Tibet,Linzhi	Jin,L et al.2013
LT079	JX068158	Linzhi Tibetan pig	Tibet,Linzhi	Jin,L et al.2013
LT080	JX068157	Linzhi Tibetan pig	Tibet,Linzhi	Jin,L et al.2013
LT081	JX068156	Linzhi Tibetan pig	Tibet,Linzhi	Jin,L et al.2013
LT082	JX068155	Linzhi Tibetan pig	Tibet,Linzhi	Jin,L et al.2013
LT083	JX068154	Linzhi Tibetan pig	Tibet,Linzhi	Jin,L et al.2013
LT084	JX068153	Linzhi Tibetan pig	Tibet,Linzhi	Jin,L et al.2013
LT085	JX068152	Linzhi Tibetan pig	Tibet,Linzhi	Jin,L et al.2013
LT086	JX068151	Linzhi Tibetan pig	Tibet,Linzhi	Jin,L et al.2013
LT087	JX068150	Linzhi Tibetan pig	Tibet,Linzhi	Jin,L et al.2013
LT088	JX068149	Linzhi Tibetan pig	Tibet,Linzhi	Jin,L et al.2013
LT089	JX068148	Linzhi Tibetan pig	Tibet,Linzhi	Jin,L et al.2013
LT090	JX068147	Linzhi Tibetan pig	Tibet,Linzhi	Jin,L et al.2013
LT091	JX068146	Linzhi Tibetan pig	Tibet,Linzhi	Jin,L et al.2013
LT092	JX068145	Linzhi Tibetan pig	Tibet,Linzhi	Jin,L et al.2013
LT093	JX068144	Linzhi Tibetan pig	Tibet,Linzhi	Jin,L et al.2013
LT094	JX068143	Linzhi Tibetan pig	Tibet,Linzhi	Jin,L et al.2013
LT095	JX068142	Linzhi Tibetan pig	Tibet,Linzhi	Jin,L et al.2013
LT096	JX068141	Linzhi Tibetan pig	Tibet,Linzhi	Jin,L et al.2013
LT097	HQ148343	Linzhi Tibetan pig	Tibet,Linzhi	Shuli Yang et al.2011
LT098	HQ148342	Linzhi Tibetan pig	Tibet,Linzhi	Shuli Yang et al.2011
LT099	HQ148341	Linzhi Tibetan pig	Tibet,Linzhi	Shuli Yang et al.2011
LT100	HQ148340	Linzhi Tibetan pig	Tibet,Linzhi	Shuli Yang et al.2011
LT101	HQ148339	Linzhi Tibetan pig	Tibet,Linzhi	Shuli Yang et al.2011
LT102	HQ148338	Linzhi Tibetan pig	Tibet,Linzhi	Shuli Yang et al.2011
LT103	HQ148337	Linzhi Tibetan pig	Tibet,Linzhi	Shuli Yang et al.2011
LT104	HQ148336	Linzhi Tibetan pig	Tibet,Linzhi	Shuli Yang et al.2011
LT105	HQ148335	Linzhi Tibetan pig	Tibet,Linzhi	Shuli Yang et al.2011
LT106	HQ148334	Linzhi Tibetan pig	Tibet,Linzhi	Shuli Yang et al.2011
LT107	HQ148333	Linzhi Tibetan pig	Tibet,Linzhi	Shuli Yang et al.2011
LT108	HQ148332	Linzhi Tibetan pig	Tibet,Linzhi	Shuli Yang et al.2011
LT109	HQ148331	Linzhi Tibetan pig	Tibet,Linzhi	Shuli Yang et al.2011
LT110	HQ148330	Linzhi Tibetan pig	Tibet,Linzhi	Shuli Yang et al.2011
LT111	HQ148329	Linzhi Tibetan pig	Tibet,Linzhi	Shuli Yang et al.2011
LT112	HQ148328	Linzhi Tibetan pig	Tibet,Linzhi	Shuli Yang et al.2011
LT113	HQ148327	Linzhi Tibetan pig	Tibet,Linzhi	Shuli Yang et al.2011
LT114	HQ148326	Linzhi Tibetan pig	Tibet,Linzhi	Shuli Yang et al.2011
LT115	HQ148315	Linzhi Tibetan pig	Tibet,Linzhi	Shuli Yang et al.2011
LT116	HQ148314	Linzhi Tibetan pig	Tibet,Linzhi	Shuli Yang et al.2011
LT117	HQ148313	Linzhi Tibetan pig	Tibet,Linzhi	Shuli Yang et al.2011
LT118	HQ148312	Linzhi Tibetan pig	Tibet,Linzhi	Shuli Yang et al.2011
LT119	HQ148311	Linzhi Tibetan pig	Tibet,Linzhi	Shuli Yang et al.2011
LT120		Linzhi Tibetan pig	Tibet,Linzhi	This study
LT121		Linzhi Tibetan pig	Tibet,Linzhi	This study
LT122		Linzhi Tibetan pig	Tibet,Linzhi	This study
LT123		Linzhi Tibetan pig	Tibet,Linzhi	This study
LT124		Linzhi Tibetan pig	Tibet,Linzhi	This study
LT125		Linzhi Tibetan pig	Tibet,Linzhi	This study
LT126		Linzhi Tibetan pig	Tibet,Linzhi	This study
LT127		Linzhi Tibetan pig	Tibet,Linzhi	This study
LT128		Linzhi Tibetan pig	Tibet,Linzhi	This study
LT129		Linzhi Tibetan pig	Tibet,Linzhi	This study
LT130		Linzhi Tibetan pig	Tibet,Linzhi	This study
LT131		Linzhi Tibetan pig	Tibet,Linzhi	This study
LT132		Linzhi Tibetan pig	Tibet,Linzhi	This study
LT133		Linzhi Tibetan pig	Tibet,Linzhi	This study
LT134		Linzhi Tibetan pig	Tibet,Linzhi	This study
LT135		Linzhi Tibetan pig	Tibet,Linzhi	This study
LT136		Linzhi Tibetan pig	Tibet,Linzhi	This study
LT137		Linzhi Tibetan pig	Tibet,Linzhi	This study
LT138		Linzhi Tibetan pig	Tibet,Linzhi	This study
LT139		Linzhi Tibetan pig	Tibet,Linzhi	This study
LT140		Linzhi Tibetan pig	Tibet,Linzhi	This study
LT141		Linzhi Tibetan pig	Tibet,Linzhi	This study
LT142		Linzhi Tibetan pig	Tibet,Linzhi	This study
LT143		Linzhi Tibetan pig	Tibet,Linzhi	This study
LT144		Linzhi Tibetan pig	Tibet,Linzhi	This study
LT145		Linzhi Tibetan pig	Tibet,Linzhi	This study
LT146		Linzhi Tibetan pig	Tibet,Linzhi	This study
LT147		Linzhi Tibetan pig	Tibet,Linzhi	This study
LT148		Linzhi Tibetan pig	Tibet,Linzhi	This study
LT149		Linzhi Tibetan pig	Tibet,Linzhi	This study
LT150		Linzhi Tibetan pig	Tibet,Linzhi	This study
LT151		Linzhi Tibetan pig	Tibet,Linzhi	This study
LT152		Linzhi Tibetan pig	Tibet,Linzhi	This study
LT153		Linzhi Tibetan pig	Tibet,Linzhi	This study
LT154		Linzhi Tibetan pig	Tibet,Linzhi	This study
LT155		Linzhi Tibetan pig	Tibet,Linzhi	This study
LT156		Linzhi Tibetan pig	Tibet,Linzhi	This study
LT157		Linzhi Tibetan pig	Tibet,Linzhi	This study
LT158		Linzhi Tibetan pig	Tibet,Linzhi	This study
LT159		Linzhi Tibetan pig	Tibet,Linzhi	This study
LT160		Linzhi Tibetan pig	Tibet,Linzhi	This study
LT161		Linzhi Tibetan pig	Tibet,Linzhi	This study
LT162		Linzhi Tibetan pig	Tibet,Linzhi	This study
LT163		Linzhi Tibetan pig	Tibet,Linzhi	This study
LT164		Linzhi Tibetan pig	Tibet,Linzhi	This study
LT165		Linzhi Tibetan pig	Tibet,Linzhi	This study
LT166		Linzhi Tibetan pig	Tibet,Linzhi	This study
LT167		Linzhi Tibetan pig	Tibet,Linzhi	This study
LT168		Linzhi Tibetan pig	Tibet,Linzhi	This study
LT169		Linzhi Tibetan pig	Tibet,Linzhi	This study
LT170		Linzhi Tibetan pig	Tibet,Linzhi	This study
LT171		Linzhi Tibetan pig	Tibet,Linzhi	This study
LT172		Linzhi Tibetan pig	Tibet,Linzhi	This study
LT173		Linzhi Tibetan pig	Tibet,Linzhi	This study
LT174		Linzhi Tibetan pig	Tibet,Linzhi	This study
LT175		Linzhi Tibetan pig	Tibet,Linzhi	This study
LT176		Linzhi Tibetan pig	Tibet,Linzhi	This study
LT177		Linzhi Tibetan pig	Tibet,Linzhi	This study
LT178		Linzhi Tibetan pig	Tibet,Linzhi	This study
LT179		Linzhi Tibetan pig	Tibet,Linzhi	This study
LT180		Linzhi Tibetan pig	Tibet,Linzhi	This study
LT181		Linzhi Tibetan pig	Tibet,Linzhi	This study
LT182		Linzhi Tibetan pig	Tibet,Linzhi	This study
LT183		Linzhi Tibetan pig	Tibet,Linzhi	This study
LT184		Linzhi Tibetan pig	Tibet,Linzhi	This study
LT185		Linzhi Tibetan pig	Tibet,Linzhi	This study
LT186		Linzhi Tibetan pig	Tibet,Linzhi	This study
LT187		Linzhi Tibetan pig	Tibet,Linzhi	This study
LT188		Linzhi Tibetan pig	Tibet,Linzhi	This study
LT189		Linzhi Tibetan pig	Tibet,Linzhi	This study
LT190		Linzhi Tibetan pig	Tibet,Linzhi	This study
LT191		Linzhi Tibetan pig	Tibet,Linzhi	This study
LT192		Linzhi Tibetan pig	Tibet,Linzhi	This study
LT193		Linzhi Tibetan pig	Tibet,Linzhi	This study
LT194		Linzhi Tibetan pig	Tibet,Linzhi	This study
LT195		Linzhi Tibetan pig	Tibet,Linzhi	This study
LT196		Linzhi Tibetan pig	Tibet,Linzhi	This study
LT197		Linzhi Tibetan pig	Tibet,Linzhi	This study
LT198		Linzhi Tibetan pig	Tibet,Linzhi	This study
LT199		Linzhi Tibetan pig	Tibet,Linzhi	This study
LT200		Linzhi Tibetan pig	Tibet,Linzhi	This study
LT201		Linzhi Tibetan pig	Tibet,Linzhi	This study
LT202		Linzhi Tibetan pig	Tibet,Linzhi	This study
LT203		Linzhi Tibetan pig	Tibet,Linzhi	This study
LT204		Linzhi Tibetan pig	Tibet,Linzhi	This study
LT205		Linzhi Tibetan pig	Tibet,Linzhi	This study
LT206		Linzhi Tibetan pig	Tibet,Linzhi	This study
LT207		Linzhi Tibetan pig	Tibet,Linzhi	This study
LT208		Linzhi Tibetan pig	Tibet,Linzhi	This study
LT209		Linzhi Tibetan pig	Tibet,Linzhi	This study
LT210		Linzhi Tibetan pig	Tibet,Linzhi	This study
LT211		Linzhi Tibetan pig	Tibet,Linzhi	This study
LT212		Linzhi Tibetan pig	Tibet,Linzhi	This study
LT213		Linzhi Tibetan pig	Tibet,Linzhi	This study
LT214		Linzhi Tibetan pig	Tibet,Linzhi	This study
LT215		Linzhi Tibetan pig	Tibet,Linzhi	This study
LT216		Linzhi Tibetan pig	Tibet,Linzhi	This study
LT217		Linzhi Tibetan pig	Tibet,Linzhi	This study
LT218		Linzhi Tibetan pig	Tibet,Linzhi	This study
LT219		Linzhi Tibetan pig	Tibet,Linzhi	This study
LT220		Linzhi Tibetan pig	Tibet,Linzhi	This study
LT221		Linzhi Tibetan pig	Tibet,Linzhi	This study
LT222		Linzhi Tibetan pig	Tibet,Linzhi	This study
LT223		Linzhi Tibetan pig	Tibet,Linzhi	This study
LT224		Linzhi Tibetan pig	Tibet,Linzhi	This study
LT225		Linzhi Tibetan pig	Tibet,Linzhi	This study
LT226		Linzhi Tibetan pig	Tibet,Linzhi	This study
LT227		Linzhi Tibetan pig	Tibet,Linzhi	This study
LT228		Linzhi Tibetan pig	Tibet,Linzhi	This study
LT229		Linzhi Tibetan pig	Tibet,Linzhi	This study
LT230		Linzhi Tibetan pig	Tibet,Linzhi	This study
LT231		Linzhi Tibetan pig	Tibet,Linzhi	This study
LT232		Linzhi Tibetan pig	Tibet,Linzhi	This study
LT233		Linzhi Tibetan pig	Tibet,Linzhi	This study
LT234		Linzhi Tibetan pig	Tibet,Linzhi	This study
LT235		Linzhi Tibetan pig	Tibet,Linzhi	This study
LT236		Linzhi Tibetan pig	Tibet,Linzhi	This study
LT237		Linzhi Tibetan pig	Tibet,Linzhi	This study
LT238		Linzhi Tibetan pig	Tibet,Linzhi	This study
LT239		Linzhi Tibetan pig	Tibet,Linzhi	This study
LT240		Linzhi Tibetan pig	Tibet,Linzhi	This study
LT241		Linzhi Tibetan pig	Tibet,Linzhi	This study
ST001	DQ152892.2	Shannan Tibetan pig	Tibet, Shannan	Fang,M et al.2006
ST002	DQ152874.2	Shannan Tibetan pig	Tibet, Shannan	Fang,M et al.2006
ST003	DQ152868.2	Shannan Tibetan pig	Tibet, Shannan	Fang,M et al.2006
ST004	DQ379102.2	Shannan Tibetan pig	Tibet, Shannan	Fang,M et al.2006
ST005	DQ379101.2	Shannan Tibetan pig	Tibet, Shannan	Fang,M et al.2006
ST006	AF486856	Shannan Tibetan pig	Tibet, Shannan	Yang,J et al.2004
ST007	AY486116	Shannan Tibetan pig	Tibet, Shannan	Gongora,J et al.2004
ST008	AY463062	Shannan Tibetan pig	Tibet, Shannan	Gongora,J et al.2004
ST009	HQ148366	Shannan Tibetan pig	Tibet, Shannan	Shuli Yang et al.2011
ST010	HQ148365	Shannan Tibetan pig	Tibet, Shannan	Shuli Yang et al.2011
ST011	HQ148364	Shannan Tibetan pig	Tibet, Shannan	Shuli Yang et al.2011
ST012	HQ148363	Shannan Tibetan pig	Tibet, Shannan	Shuli Yang et al.2011
ST013	HQ148362	Shannan Tibetan pig	Tibet, Shannan	Shuli Yang et al.2011
ST014	HQ148361	Shannan Tibetan pig	Tibet, Shannan	Shuli Yang et al.2011
ST015	HQ148360	Shannan Tibetan pig	Tibet, Shannan	Shuli Yang et al.2011
ST016	HQ148359	Shannan Tibetan pig	Tibet, Shannan	Shuli Yang et al.2011
ST017	HQ148358	Shannan Tibetan pig	Tibet, Shannan	Shuli Yang et al.2011
ST018	HQ148357	Shannan Tibetan pig	Tibet, Shannan	Shuli Yang et al.2011
ST019	HQ148356	Shannan Tibetan pig	Tibet, Shannan	Shuli Yang et al.2011
ST020		Shannan Tibetan pig	Tibet, Shannan	This study
ST021		Shannan Tibetan pig	Tibet, Shannan	This study
ST022		Shannan Tibetan pig	Tibet, Shannan	This study
ST023		Shannan Tibetan pig	Tibet, Shannan	This study
ST024		Shannan Tibetan pig	Tibet, Shannan	This study
ST025		Shannan Tibetan pig	Tibet, Shannan	This study
ST026		Shannan Tibetan pig	Tibet, Shannan	This study
ST027		Shannan Tibetan pig	Tibet, Shannan	This study
ST028		Shannan Tibetan pig	Tibet, Shannan	This study
ST029		Shannan Tibetan pig	Tibet, Shannan	This study
ST030		Shannan Tibetan pig	Tibet, Shannan	This study
ST031		Shannan Tibetan pig	Tibet, Shannan	This study
ST032		Shannan Tibetan pig	Tibet, Shannan	This study
ST033		Shannan Tibetan pig	Tibet, Shannan	This study
ST034		Shannan Tibetan pig	Tibet, Shannan	This study
ST035		Shannan Tibetan pig	Tibet, Shannan	This study
ST036		Shannan Tibetan pig	Tibet, Shannan	This study
ST037		Shannan Tibetan pig	Tibet, Shannan	This study
ST038		Shannan Tibetan pig	Tibet, Shannan	This study
ST039		Shannan Tibetan pig	Tibet, Shannan	This study
ST040		Shannan Tibetan pig	Tibet, Shannan	This study
ST041		Shannan Tibetan pig	Tibet, Shannan	This study
ST042		Shannan Tibetan pig	Tibet, Shannan	This study
ST043		Shannan Tibetan pig	Tibet, Shannan	This study
ST044		Shannan Tibetan pig	Tibet, Shannan	This study
ST045		Shannan Tibetan pig	Tibet, Shannan	This study
ST046		Shannan Tibetan pig	Tibet, Shannan	This study
ST047		Shannan Tibetan pig	Tibet, Shannan	This study
ST048		Shannan Tibetan pig	Tibet, Shannan	This study
ST049		Shannan Tibetan pig	Tibet, Shannan	This study
ST050		Shannan Tibetan pig	Tibet, Shannan	This study
ST051		Shannan Tibetan pig	Tibet, Shannan	This study
ST052		Shannan Tibetan pig	Tibet, Shannan	This study
ST053		Shannan Tibetan pig	Tibet, Shannan	This study
ST054		Shannan Tibetan pig	Tibet, Shannan	This study
ST055		Shannan Tibetan pig	Tibet, Shannan	This study
ST056		Shannan Tibetan pig	Tibet, Shannan	This study
ST057		Shannan Tibetan pig	Tibet, Shannan	This study
ST058		Shannan Tibetan pig	Tibet, Shannan	This study
ST059		Shannan Tibetan pig	Tibet, Shannan	This study
ST060		Shannan Tibetan pig	Tibet, Shannan	This study
ST061		Shannan Tibetan pig	Tibet, Shannan	This study
ST062		Shannan Tibetan pig	Tibet, Shannan	This study
ST063		Shannan Tibetan pig	Tibet, Shannan	This study
ST064		Shannan Tibetan pig	Tibet, Shannan	This study
ST065		Shannan Tibetan pig	Tibet, Shannan	This study
ST066		Shannan Tibetan pig	Tibet, Shannan	This study
ST067		Shannan Tibetan pig	Tibet, Shannan	This study
ST068		Shannan Tibetan pig	Tibet, Shannan	This study
ST069		Shannan Tibetan pig	Tibet, Shannan	This study
ST070		Shannan Tibetan pig	Tibet, Shannan	This study
ST071		Shannan Tibetan pig	Tibet, Shannan	This study
ST072		Shannan Tibetan pig	Tibet, Shannan	This study
ST073		Shannan Tibetan pig	Tibet, Shannan	This study
ST074		Shannan Tibetan pig	Tibet, Shannan	This study
ST075		Shannan Tibetan pig	Tibet, Shannan	This study
ST076		Shannan Tibetan pig	Tibet, Shannan	This study
ST077		Shannan Tibetan pig	Tibet, Shannan	This study
ST078		Shannan Tibetan pig	Tibet, Shannan	This study
ST079		Shannan Tibetan pig	Tibet, Shannan	This study
ST080		Shannan Tibetan pig	Tibet, Shannan	This study
ST081		Shannan Tibetan pig	Tibet, Shannan	This study
ST082		Shannan Tibetan pig	Tibet, Shannan	This study
ST083		Shannan Tibetan pig	Tibet, Shannan	This study
ST084		Shannan Tibetan pig	Tibet, Shannan	This study
ST085		Shannan Tibetan pig	Tibet, Shannan	This study
ST086		Shannan Tibetan pig	Tibet, Shannan	This study
ST087		Shannan Tibetan pig	Tibet, Shannan	This study
ST088		Shannan Tibetan pig	Tibet, Shannan	This study
ST089		Shannan Tibetan pig	Tibet, Shannan	This study
ST090		Shannan Tibetan pig	Tibet, Shannan	This study
ST091		Shannan Tibetan pig	Tibet, Shannan	This study
CT001	HQ148410	Changdu Tibetan pig	Tibet,Changdu	Shuli Yang et al.2011
CT002	HQ148409	Changdu Tibetan pig	Tibet,Changdu	Shuli Yang et al.2011
CT003	HQ148408	Changdu Tibetan pig	Tibet,Changdu	Shuli Yang et al.2011
CT004	HQ148407	Changdu Tibetan pig	Tibet,Changdu	Shuli Yang et al.2011
CT005	HQ148406	Changdu Tibetan pig	Tibet,Changdu	Shuli Yang et al.2011
CT006	HQ148405	Changdu Tibetan pig	Tibet,Changdu	Shuli Yang et al.2011
CT007	HQ148404	Changdu Tibetan pig	Tibet,Changdu	Shuli Yang et al.2011
CT008	HQ148403	Changdu Tibetan pig	Tibet,Changdu	Shuli Yang et al.2011
CT009	HQ148402	Changdu Tibetan pig	Tibet,Changdu	Shuli Yang et al.2011
CT010	HQ148401	Changdu Tibetan pig	Tibet,Changdu	Shuli Yang et al.2011
CT011	HQ148400	Changdu Tibetan pig	Tibet,Changdu	Shuli Yang et al.2011
CT012	HQ148399	Changdu Tibetan pig	Tibet,Changdu	Shuli Yang et al.2011
CT013	HQ148398	Changdu Tibetan pig	Tibet,Changdu	Shuli Yang et al.2011
CT014	HQ148397	Changdu Tibetan pig	Tibet,Changdu	Shuli Yang et al.2011
CT015	HQ148396	Changdu Tibetan pig	Tibet,Changdu	Shuli Yang et al.2011
CT016	HQ148395-HQ	Changdu Tibetan pig	Tibet,Changdu	Shuli Yang et al.2011
CT017	HQ148394	Changdu Tibetan pig	Tibet,Changdu	Shuli Yang et al.2011
CT018		Changdu Tibetan pig	Tibet,Changdu	This study
CT019		Changdu Tibetan pig	Tibet,Changdu	This study
CT020		Changdu Tibetan pig	Tibet,Changdu	This study
CT021		Changdu Tibetan pig	Tibet,Changdu	This study
CT022		Changdu Tibetan pig	Tibet,Changdu	This study
CT023		Changdu Tibetan pig	Tibet,Changdu	This study
CT024		Changdu Tibetan pig	Tibet,Changdu	This study
CT025		Changdu Tibetan pig	Tibet,Changdu	This study
CT026		Changdu Tibetan pig	Tibet,Changdu	This study
CT027		Changdu Tibetan pig	Tibet,Changdu	This study
CT028		Changdu Tibetan pig	Tibet,Changdu	This study
CT029		Changdu Tibetan pig	Tibet,Changdu	This study
CT030		Changdu Tibetan pig	Tibet,Changdu	This study
CT031		Changdu Tibetan pig	Tibet,Changdu	This study
CT032		Changdu Tibetan pig	Tibet,Changdu	This study
CT033		Changdu Tibetan pig	Tibet,Changdu	This study
CT034		Changdu Tibetan pig	Tibet,Changdu	This study
CT035		Changdu Tibetan pig	Tibet,Changdu	This study
CT036		Changdu Tibetan pig	Tibet,Changdu	This study
CT037		Changdu Tibetan pig	Tibet,Changdu	This study
CT038		Changdu Tibetan pig	Tibet,Changdu	This study
CT039		Changdu Tibetan pig	Tibet,Changdu	This study
CT040		Changdu Tibetan pig	Tibet,Changdu	This study
CT041		Changdu Tibetan pig	Tibet,Changdu	This study
CT042		Changdu Tibetan pig	Tibet,Changdu	This study
CT043		Changdu Tibetan pig	Tibet,Changdu	This study
CT044		Changdu Tibetan pig	Tibet,Changdu	This study
CT045		Changdu Tibetan pig	Tibet,Changdu	This study
CT046		Changdu Tibetan pig	Tibet,Changdu	This study
CT047		Changdu Tibetan pig	Tibet,Changdu	This study
CT048		Changdu Tibetan pig	Tibet,Changdu	This study
CT049		Changdu Tibetan pig	Tibet,Changdu	This study
CT050		Changdu Tibetan pig	Tibet,Changdu	This study
CT051		Changdu Tibetan pig	Tibet,Changdu	This study
CT052		Changdu Tibetan pig	Tibet,Changdu	This study
CT053		Changdu Tibetan pig	Tibet,Changdu	This study
CT054		Changdu Tibetan pig	Tibet,Changdu	This study
CT055		Changdu Tibetan pig	Tibet,Changdu	This study
CT056		Changdu Tibetan pig	Tibet,Changdu	This study
CT057		Changdu Tibetan pig	Tibet,Changdu	This study
CT058		Changdu Tibetan pig	Tibet,Changdu	This study
CT059		Changdu Tibetan pig	Tibet,Changdu	This study
CT060		Changdu Tibetan pig	Tibet,Changdu	This study
CT061		Changdu Tibetan pig	Tibet,Changdu	This study
CT062		Changdu Tibetan pig	Tibet,Changdu	This study
CT063		Changdu Tibetan pig	Tibet,Changdu	This study
CT064		Changdu Tibetan pig	Tibet,Changdu	This study
CT065		Changdu Tibetan pig	Tibet,Changdu	This study
CT066		Changdu Tibetan pig	Tibet,Changdu	This study
CT067		Changdu Tibetan pig	Tibet,Changdu	This study
CT068		Changdu Tibetan pig	Tibet,Changdu	This study
CT069		Changdu Tibetan pig	Tibet,Changdu	This study
CT070		Changdu Tibetan pig	Tibet,Changdu	This study
CT071		Changdu Tibetan pig	Tibet,Changdu	This study
CT072		Changdu Tibetan pig	Tibet,Changdu	This study
CT073		Changdu Tibetan pig	Tibet,Changdu	This study
CT074		Changdu Tibetan pig	Tibet,Changdu	This study
CT075		Changdu Tibetan pig	Tibet,Changdu	This study
CT076		Changdu Tibetan pig	Tibet,Changdu	This study
CT077		Changdu Tibetan pig	Tibet,Changdu	This study
CT078		Changdu Tibetan pig	Tibet,Changdu	This study
CT079		Changdu Tibetan pig	Tibet,Changdu	This study
CT080		Changdu Tibetan pig	Tibet,Changdu	This study
CT081		Changdu Tibetan pig	Tibet,Changdu	This study
CT082		Changdu Tibetan pig	Tibet,Changdu	This study
CT083		Changdu Tibetan pig	Tibet,Changdu	This study
CT084		Changdu Tibetan pig	Tibet,Changdu	This study
CT085		Changdu Tibetan pig	Tibet,Changdu	This study
CT086		Changdu Tibetan pig	Tibet,Changdu	This study
CT087		Changdu Tibetan pig	Tibet,Changdu	This study
CT088		Changdu Tibetan pig	Tibet,Changdu	This study
CT089		Changdu Tibetan pig	Tibet,Changdu	This study
CT090		Changdu Tibetan pig	Tibet,Changdu	This study
RT001	JX068140	Rkaze Tibetan pig	Tibet,Rkaze	Jin,L et al.2013
RT002	JX068139	Rkaze Tibetan pig	Tibet,Rkaze	Jin,L et al.2013
RT003	JX068138	Rkaze Tibetan pig	Tibet,Rkaze	Jin,L et al.2013
RT004	JX068137	Rkaze Tibetan pig	Tibet,Rkaze	Jin,L et al.2013
RT005	JX068136	Rkaze Tibetan pig	Tibet,Rkaze	Jin,L et al.2013
RT006	JX068135	Rkaze Tibetan pig	Tibet,Rkaze	Jin,L et al.2013
RT007	JX068134	Rkaze Tibetan pig	Tibet,Rkaze	Jin,L et al.2013
RT008	JX068133	Rkaze Tibetan pig	Tibet,Rkaze	Jin,L et al.2013
RT009	JX068132	Rkaze Tibetan pig	Tibet,Rkaze	Jin,L et al.2013
RT010	JX068131	Rkaze Tibetan pig	Tibet,Rkaze	Jin,L et al.2013
RT011	JX068130	Rkaze Tibetan pig	Tibet,Rkaze	Jin,L et al.2013
RT012	JX068129	Rkaze Tibetan pig	Tibet,Rkaze	Jin,L et al.2013
RT013	JX068128	Rkaze Tibetan pig	Tibet,Rkaze	Jin,L et al.2013
RT014	JX068127	Rkaze Tibetan pig	Tibet,Rkaze	Jin,L et al.2013
RT015	JX068126	Rkaze Tibetan pig	Tibet,Rkaze	Jin,L et al.2013
RT016	JX068125	Rkaze Tibetan pig	Tibet,Rkaze	Jin,L et al.2013
RT017	JX068124	Rkaze Tibetan pig	Tibet,Rkaze	Jin,L et al.2013
RT018	JX068123	Rkaze Tibetan pig	Tibet,Rkaze	Jin,L et al.2013
RT019	JX068122	Rkaze Tibetan pig	Tibet,Rkaze	Jin,L et al.2013
RT020	JX068121	Rkaze Tibetan pig	Tibet,Rkaze	Jin,L et al.2013
RT021	JX068120	Rkaze Tibetan pig	Tibet,Rkaze	Jin,L et al.2013
RT022	JX068119	Rkaze Tibetan pig	Tibet,Rkaze	Jin,L et al.2013
RT023	JX068118	Rkaze Tibetan pig	Tibet,Rkaze	Jin,L et al.2013
RT024	JX068117	Rkaze Tibetan pig	Tibet,Rkaze	Jin,L et al.2013
HT001	JX068354	Hezuo Tibetan pig	Gansu,Hezuo	Jin,L et al.2013
HT002	JX068353	Hezuo Tibetan pig	Gansu,Hezuo	Jin,L et al.2013
HT003	JX068352	Hezuo Tibetan pig	Gansu,Hezuo	Jin,L et al.2013
HT004	JX068351	Hezuo Tibetan pig	Gansu,Hezuo	Jin,L et al.2013
HT005	JX068350	Hezuo Tibetan pig	Gansu,Hezuo	Jin,L et al.2013
HT006	JX068349	Hezuo Tibetan pig	Gansu,Hezuo	Jin,L et al.2013
HT007	JX068348	Hezuo Tibetan pig	Gansu,Hezuo	Jin,L et al.2013
HT008	JX068347	Hezuo Tibetan pig	Gansu,Hezuo	Jin,L et al.2013
HT009	JX068346	Hezuo Tibetan pig	Gansu,Hezuo	Jin,L et al.2013
HT010	JX068345	Hezuo Tibetan pig	Gansu,Hezuo	Jin,L et al.2013
HT011	JX068344	Hezuo Tibetan pig	Gansu,Hezuo	Jin,L et al.2013
HT012	JX068343	Hezuo Tibetan pig	Gansu,Hezuo	Jin,L et al.2013
HT013	JX068342	Hezuo Tibetan pig	Gansu,Hezuo	Jin,L et al.2013
HT014	JX068341	Hezuo Tibetan pig	Gansu,Hezuo	Jin,L et al.2013
HT015	JX068340	Hezuo Tibetan pig	Gansu,Hezuo	Jin,L et al.2013
HT016	JX068339	Hezuo Tibetan pig	Gansu,Hezuo	Jin,L et al.2013
HT017	JX068338	Hezuo Tibetan pig	Gansu,Hezuo	Jin,L et al.2013
HT018	JX068337	Hezuo Tibetan pig	Gansu,Hezuo	Jin,L et al.2013
HT019	JX068336	Hezuo Tibetan pig	Gansu,Hezuo	Jin,L et al.2013
HT020	JX068335	Hezuo Tibetan pig	Gansu,Hezuo	Jin,L et al.2013
HT021	JX068334	Hezuo Tibetan pig	Gansu,Hezuo	Jin,L et al.2013
HT022	JX068333	Hezuo Tibetan pig	Gansu,Hezuo	Jin,L et al.2013
HT023	JX068332	Hezuo Tibetan pig	Gansu,Hezuo	Jin,L et al.2013
HT024	JX068331	Hezuo Tibetan pig	Gansu,Hezuo	Jin,L et al.2013
HT025	JX068330	Hezuo Tibetan pig	Gansu,Hezuo	Jin,L et al.2013
HT026	JX068329	Hezuo Tibetan pig	Gansu,Hezuo	Jin,L et al.2013
HT027	JX068328	Hezuo Tibetan pig	Gansu,Hezuo	Jin,L et al.2013
HT028	JX068327	Hezuo Tibetan pig	Gansu,Hezuo	Jin,L et al.2013
HT029	JX068326	Hezuo Tibetan pig	Gansu,Hezuo	Jin,L et al.2013
HT030	JX068325	Hezuo Tibetan pig	Gansu,Hezuo	Jin,L et al.2013
HT031	JX068324	Hezuo Tibetan pig	Gansu,Hezuo	Jin,L et al.2013
HT032	JX068323	Hezuo Tibetan pig	Gansu,Hezuo	Jin,L et al.2013
HT033	JX068322	Hezuo Tibetan pig	Gansu,Hezuo	Jin,L et al.2013
HT034	JX068321	Hezuo Tibetan pig	Gansu,Hezuo	Jin,L et al.2013
HT035	JX068320	Hezuo Tibetan pig	Gansu,Hezuo	Jin,L et al.2013
HT036	JX068319	Hezuo Tibetan pig	Gansu,Hezuo	Jin,L et al.2013
HT037	JX068318	Hezuo Tibetan pig	Gansu,Hezuo	Jin,L et al.2013
HT038	JX068317	Hezuo Tibetan pig	Gansu,Hezuo	Jin,L et al.2013
HT039	JX068316	Hezuo Tibetan pig	Gansu,Hezuo	Jin,L et al.2013
HT040	JX068315	Hezuo Tibetan pig	Gansu,Hezuo	Jin,L et al.2013
HT041	JX068314	Hezuo Tibetan pig	Gansu,Hezuo	Jin,L et al.2013
HT042	JX068313	Hezuo Tibetan pig	Gansu,Hezuo	Jin,L et al.2013
HT043	JX068312	Hezuo Tibetan pig	Gansu,Hezuo	Jin,L et al.2013
HT044	JX068311	Hezuo Tibetan pig	Gansu,Hezuo	Jin,L et al.2013
HT045	JX068310	Hezuo Tibetan pig	Gansu,Hezuo	Jin,L et al.2013
HT046	JX068309	Hezuo Tibetan pig	Gansu,Hezuo	Jin,L et al.2013
HT047	JX068308	Hezuo Tibetan pig	Gansu,Hezuo	Jin,L et al.2013
HT048	JX068307	Hezuo Tibetan pig	Gansu,Hezuo	Jin,L et al.2013
HT049	HQ148393	Hezuo Tibetan pig	Gansu,Hezuo	Shuli Yang et al.2011
HT050	HQ148392	Hezuo Tibetan pig	Gansu,Hezuo	Shuli Yang et al.2011
HT051	HQ148391	Hezuo Tibetan pig	Gansu,Hezuo	Shuli Yang et al.2011
HT052	HQ148390	Hezuo Tibetan pig	Gansu,Hezuo	Shuli Yang et al.2011
HT053	HQ148389	Hezuo Tibetan pig	Gansu,Hezuo	Shuli Yang et al.2011
HT054	HQ148388	Hezuo Tibetan pig	Gansu,Hezuo	Shuli Yang et al.2011
HT055	HQ148387	Hezuo Tibetan pig	Gansu,Hezuo	Shuli Yang et al.2011
HT056	HQ148386	Hezuo Tibetan pig	Gansu,Hezuo	Shuli Yang et al.2011
HT057	HQ148385	Hezuo Tibetan pig	Gansu,Hezuo	Shuli Yang et al.2011
HT058	HQ148384	Hezuo Tibetan pig	Gansu,Hezuo	Shuli Yang et al.2011
HT059	HQ148383	Hezuo Tibetan pig	Gansu,Hezuo	Shuli Yang et al.2011
HT060	HQ148382	Hezuo Tibetan pig	Gansu,Hezuo	Shuli Yang et al.2011
HT061	HQ148381	Hezuo Tibetan pig	Gansu,Hezuo	Shuli Yang et al.2011
HT062	HQ148380	Hezuo Tibetan pig	Gansu,Hezuo	Shuli Yang et al.2011
HT063	HQ148379	Hezuo Tibetan pig	Gansu,Hezuo	Shuli Yang et al.2011
HT064	HQ148378	Hezuo Tibetan pig	Gansu,Hezuo	Shuli Yang et al.2011
HT065	HQ148377	Hezuo Tibetan pig	Gansu,Hezuo	Shuli Yang et al.2011
HT066	HQ148376	Hezuo Tibetan pig	Gansu,Hezuo	Shuli Yang et al.2011
HT067	HQ148375	Hezuo Tibetan pig	Gansu,Hezuo	Shuli Yang et al.2011
HT068	HQ148374	Hezuo Tibetan pig	Gansu,Hezuo	Shuli Yang et al.2011
HT069	HQ148373	Hezuo Tibetan pig	Gansu,Hezuo	Shuli Yang et al.2011
HT070	HQ148372	Hezuo Tibetan pig	Gansu,Hezuo	Shuli Yang et al.2011
HT071	HQ148371	Hezuo Tibetan pig	Gansu,Hezuo	Shuli Yang et al.2011
HT072	HQ148370	Hezuo Tibetan pig	Gansu,Hezuo	Shuli Yang et al.2011
HT073	HQ148369	Hezuo Tibetan pig	Gansu,Hezuo	Shuli Yang et al.2011
HT074	HQ148368	Hezuo Tibetan pig	Gansu,Hezuo	Shuli Yang et al.2011
HT075	HQ148367	Hezuo Tibetan pig	Gansu,Hezuo	Shuli Yang et al.2011
HT076		Hezuo Tibetan pig	Gansu,Hezuo	This study
HT079		Hezuo Tibetan pig	Gansu,Hezuo	This study
HT082		Hezuo Tibetan pig	Gansu,Hezuo	This study
HT084		Hezuo Tibetan pig	Gansu,Hezuo	This study
HT090		Hezuo Tibetan pig	Gansu,Hezuo	This study
HT091		Hezuo Tibetan pig	Gansu,Hezuo	This study
HT093		Hezuo Tibetan pig	Gansu,Hezuo	This study
HT096		Hezuo Tibetan pig	Gansu,Hezuo	This study
HT097		Hezuo Tibetan pig	Gansu,Hezuo	This study
HT099		Hezuo Tibetan pig	Gansu,Hezuo	This study
HT100		Hezuo Tibetan pig	Gansu,Hezuo	This study
HT101		Hezuo Tibetan pig	Gansu,Hezuo	This study
HT102		Hezuo Tibetan pig	Gansu,Hezuo	This study
HT105		Hezuo Tibetan pig	Gansu,Hezuo	This study
HT106		Hezuo Tibetan pig	Gansu,Hezuo	This study
HT107		Hezuo Tibetan pig	Gansu,Hezuo	This study
HT112		Hezuo Tibetan pig	Gansu,Hezuo	This study
HT115		Hezuo Tibetan pig	Gansu,Hezuo	This study
HT118		Hezuo Tibetan pig	Gansu,Hezuo	This study
HT120		Hezuo Tibetan pig	Gansu,Hezuo	This study
HT122		Hezuo Tibetan pig	Gansu,Hezuo	This study
HT125		Hezuo Tibetan pig	Gansu,Hezuo	This study
HT126		Hezuo Tibetan pig	Gansu,Hezuo	This study
HT127		Hezuo Tibetan pig	Gansu,Hezuo	This study
HT128		Hezuo Tibetan pig	Gansu,Hezuo	This study
HT129		Hezuo Tibetan pig	Gansu,Hezuo	This study
HT131		Hezuo Tibetan pig	Gansu,Hezuo	This study
HT133		Hezuo Tibetan pig	Gansu,Hezuo	This study
HT134		Hezuo Tibetan pig	Gansu,Hezuo	This study
HT135		Hezuo Tibetan pig	Gansu,Hezuo	This study
HT136		Hezuo Tibetan pig	Gansu,Hezuo	This study
HT137		Hezuo Tibetan pig	Gansu,Hezuo	This study
HT138		Hezuo Tibetan pig	Gansu,Hezuo	This study
HT139		Hezuo Tibetan pig	Gansu,Hezuo	This study
HT140		Hezuo Tibetan pig	Gansu,Hezuo	This study
HT141		Hezuo Tibetan pig	Gansu,Hezuo	This study
HT142		Hezuo Tibetan pig	Gansu,Hezuo	This study
HT143		Hezuo Tibetan pig	Gansu,Hezuo	This study
HT144		Hezuo Tibetan pig	Gansu,Hezuo	This study
HT145		Hezuo Tibetan pig	Gansu,Hezuo	This study
HT146		Hezuo Tibetan pig	Gansu,Hezuo	This study
HT147		Hezuo Tibetan pig	Gansu,Hezuo	This study
HT148		Hezuo Tibetan pig	Gansu,Hezuo	This study
HT149		Hezuo Tibetan pig	Gansu,Hezuo	This study
HT150		Hezuo Tibetan pig	Gansu,Hezuo	This study
HT151		Hezuo Tibetan pig	Gansu,Hezuo	This study
HT152		Hezuo Tibetan pig	Gansu,Hezuo	This study
HT153		Hezuo Tibetan pig	Gansu,Hezuo	This study
HT154		Hezuo Tibetan pig	Gansu,Hezuo	This study
HT155		Hezuo Tibetan pig	Gansu,Hezuo	This study
HT156		Hezuo Tibetan pig	Gansu,Hezuo	This study
HT157		Hezuo Tibetan pig	Gansu,Hezuo	This study
HT158		Hezuo Tibetan pig	Gansu,Hezuo	This study
HT159		Hezuo Tibetan pig	Gansu,Hezuo	This study
HT161		Hezuo Tibetan pig	Gansu,Hezuo	This study
HT162		Hezuo Tibetan pig	Gansu,Hezuo	This study
HT164		Hezuo Tibetan pig	Gansu,Hezuo	This study
HT166		Hezuo Tibetan pig	Gansu,Hezuo	This study
HT168		Hezuo Tibetan pig	Gansu,Hezuo	This study
HT169		Hezuo Tibetan pig	Gansu,Hezuo	This study
HT172		Hezuo Tibetan pig	Gansu,Hezuo	This study
HT173		Hezuo Tibetan pig	Gansu,Hezuo	This study
HT174		Hezuo Tibetan pig	Gansu,Hezuo	This study
HT175		Hezuo Tibetan pig	Gansu,Hezuo	This study
HT177		Hezuo Tibetan pig	Gansu,Hezuo	This study
HT179		Hezuo Tibetan pig	Gansu,Hezuo	This study
HT183		Hezuo Tibetan pig	Gansu,Hezuo	This study
HT184		Hezuo Tibetan pig	Gansu,Hezuo	This study
HT185		Hezuo Tibetan pig	Gansu,Hezuo	This study
HT186		Hezuo Tibetan pig	Gansu,Hezuo	This study
HT187		Hezuo Tibetan pig	Gansu,Hezuo	This study
HT188		Hezuo Tibetan pig	Gansu,Hezuo	This study
HT190		Hezuo Tibetan pig	Gansu,Hezuo	This study
HT196		Hezuo Tibetan pig	Gansu,Hezuo	This study
HT197		Hezuo Tibetan pig	Gansu,Hezuo	This study
HT198		Hezuo Tibetan pig	Gansu,Hezuo	This study
HT199		Hezuo Tibetan pig	Gansu,Hezuo	This study
HT200		Hezuo Tibetan pig	Gansu,Hezuo	This study
HT201		Hezuo Tibetan pig	Gansu,Hezuo	This study
HT202		Hezuo Tibetan pig	Gansu,Hezuo	This study
HT203		Hezuo Tibetan pig	Gansu,Hezuo	This study
HT205		Hezuo Tibetan pig	Gansu,Hezuo	This study
HT206		Hezuo Tibetan pig	Gansu,Hezuo	This study
HT207		Hezuo Tibetan pig	Gansu,Hezuo	This study
HT208		Hezuo Tibetan pig	Gansu,Hezuo	This study
HT209		Hezuo Tibetan pig	Gansu,Hezuo	This study
HT211		Hezuo Tibetan pig	Gansu,Hezuo	This study
HT214		Hezuo Tibetan pig	Gansu,Hezuo	This study
HT215		Hezuo Tibetan pig	Gansu,Hezuo	This study
HT216		Hezuo Tibetan pig	Gansu,Hezuo	This study
HT217		Hezuo Tibetan pig	Gansu,Hezuo	This study
HT222		Hezuo Tibetan pig	Gansu,Hezuo	This study
HT223		Hezuo Tibetan pig	Gansu,Hezuo	This study
HT224		Hezuo Tibetan pig	Gansu,Hezuo	This study
HT225		Hezuo Tibetan pig	Gansu,Hezuo	This study
HT226		Hezuo Tibetan pig	Gansu,Hezuo	This study
HT230		Hezuo Tibetan pig	Gansu,Hezuo	This study
HT231		Hezuo Tibetan pig	Gansu,Hezuo	This study
HT232		Hezuo Tibetan pig	Gansu,Hezuo	This study
HT233		Hezuo Tibetan pig	Gansu,Hezuo	This study
HT234		Hezuo Tibetan pig	Gansu,Hezuo	This study
HT236		Hezuo Tibetan pig	Gansu,Hezuo	This study
HT240		Hezuo Tibetan pig	Gansu,Hezuo	This study
HT242		Hezuo Tibetan pig	Gansu,Hezuo	This study
HT243		Hezuo Tibetan pig	Gansu,Hezuo	This study
HT245		Hezuo Tibetan pig	Gansu,Hezuo	This study
HT247		Hezuo Tibetan pig	Gansu,Hezuo	This study
HT250		Hezuo Tibetan pig	Gansu,Hezuo	This study
HT251		Hezuo Tibetan pig	Gansu,Hezuo	This study
HT252		Hezuo Tibetan pig	Gansu,Hezuo	This study
HT257		Hezuo Tibetan pig	Gansu,Hezuo	This study
HT259		Hezuo Tibetan pig	Gansu,Hezuo	This study
HT262		Hezuo Tibetan pig	Gansu,Hezuo	This study
HT267		Hezuo Tibetan pig	Gansu,Hezuo	This study
HT268		Hezuo Tibetan pig	Gansu,Hezuo	This study
HT273		Hezuo Tibetan pig	Gansu,Hezuo	This study
HT275		Hezuo Tibetan pig	Gansu,Hezuo	This study
HT276		Hezuo Tibetan pig	Gansu,Hezuo	This study
HT277		Hezuo Tibetan pig	Gansu,Hezuo	This study
HT279		Hezuo Tibetan pig	Gansu,Hezuo	This study
HT280		Hezuo Tibetan pig	Gansu,Hezuo	This study
HT281		Hezuo Tibetan pig	Gansu,Hezuo	This study
HT283		Hezuo Tibetan pig	Gansu,Hezuo	This study
HT284		Hezuo Tibetan pig	Gansu,Hezuo	This study
HT285		Hezuo Tibetan pig	Gansu,Hezuo	This study
HT289		Hezuo Tibetan pig	Gansu,Hezuo	This study
HT290		Hezuo Tibetan pig	Gansu,Hezuo	This study
HT292		Hezuo Tibetan pig	Gansu,Hezuo	This study
HT297		Hezuo Tibetan pig	Gansu,Hezuo	This study
HT298		Hezuo Tibetan pig	Gansu,Hezuo	This study
HT299		Hezuo Tibetan pig	Gansu,Hezuo	This study
HT303		Hezuo Tibetan pig	Gansu,Hezuo	This study
HT304		Hezuo Tibetan pig	Gansu,Hezuo	This study
HT307		Hezuo Tibetan pig	Gansu,Hezuo	This study
HT310		Hezuo Tibetan pig	Gansu,Hezuo	This study
HT311		Hezuo Tibetan pig	Gansu,Hezuo	This study
HT314		Hezuo Tibetan pig	Gansu,Hezuo	This study
HT315		Hezuo Tibetan pig	Gansu,Hezuo	This study
HT316		Hezuo Tibetan pig	Gansu,Hezuo	This study
HT317		Hezuo Tibetan pig	Gansu,Hezuo	This study
HT318		Hezuo Tibetan pig	Gansu,Hezuo	This study
HT319		Hezuo Tibetan pig	Gansu,Hezuo	This study
HT320		Hezuo Tibetan pig	Gansu,Hezuo	This study
HT321		Hezuo Tibetan pig	Gansu,Hezuo	This study
HT322		Hezuo Tibetan pig	Gansu,Hezuo	This study
HT323		Hezuo Tibetan pig	Gansu,Hezuo	This study
HT324		Hezuo Tibetan pig	Gansu,Hezuo	This study
HT325		Hezuo Tibetan pig	Gansu,Hezuo	This study
HT326		Hezuo Tibetan pig	Gansu,Hezuo	This study
HT327		Hezuo Tibetan pig	Gansu,Hezuo	This study
HT328		Hezuo Tibetan pig	Gansu,Hezuo	This study
HT329		Hezuo Tibetan pig	Gansu,Hezuo	This study
HT330		Hezuo Tibetan pig	Gansu,Hezuo	This study
HT331		Hezuo Tibetan pig	Gansu,Hezuo	This study
HT333		Hezuo Tibetan pig	Gansu,Hezuo	This study
HT336		Hezuo Tibetan pig	Gansu,Hezuo	This study
HT337		Hezuo Tibetan pig	Gansu,Hezuo	This study
HT339		Hezuo Tibetan pig	Gansu,Hezuo	This study
HT340		Hezuo Tibetan pig	Gansu,Hezuo	This study
HT341		Hezuo Tibetan pig	Gansu,Hezuo	This study
HT342		Hezuo Tibetan pig	Gansu,Hezuo	This study
HT343		Hezuo Tibetan pig	Gansu,Hezuo	This study
HT344		Hezuo Tibetan pig	Gansu,Hezuo	This study
HT346		Hezuo Tibetan pig	Gansu,Hezuo	This study
HT347		Hezuo Tibetan pig	Gansu,Hezuo	This study
HT348		Hezuo Tibetan pig	Gansu,Hezuo	This study
HT350		Hezuo Tibetan pig	Gansu,Hezuo	This study
HT351		Hezuo Tibetan pig	Gansu,Hezuo	This study
HT353		Hezuo Tibetan pig	Gansu,Hezuo	This study
HT355		Hezuo Tibetan pig	Gansu,Hezuo	This study
HT356		Hezuo Tibetan pig	Gansu,Hezuo	This study
HT359		Hezuo Tibetan pig	Gansu,Hezuo	This study
HT360		Hezuo Tibetan pig	Gansu,Hezuo	This study
HT361		Hezuo Tibetan pig	Gansu,Hezuo	This study
HT362		Hezuo Tibetan pig	Gansu,Hezuo	This study
HT364		Hezuo Tibetan pig	Gansu,Hezuo	This study
HT365		Hezuo Tibetan pig	Gansu,Hezuo	This study
HT367		Hezuo Tibetan pig	Gansu,Hezuo	This study
HT368		Hezuo Tibetan pig	Gansu,Hezuo	This study
HT369		Hezuo Tibetan pig	Gansu,Hezuo	This study
HT373		Hezuo Tibetan pig	Gansu,Hezuo	This study
HT375		Hezuo Tibetan pig	Gansu,Hezuo	This study
HT376		Hezuo Tibetan pig	Gansu,Hezuo	This study
HT377		Hezuo Tibetan pig	Gansu,Hezuo	This study
HT378		Hezuo Tibetan pig	Gansu,Hezuo	This study
HT379		Hezuo Tibetan pig	Gansu,Hezuo	This study
HT380		Hezuo Tibetan pig	Gansu,Hezuo	This study
HT381		Hezuo Tibetan pig	Gansu,Hezuo	This study
HT382		Hezuo Tibetan pig	Gansu,Hezuo	This study
HT383		Hezuo Tibetan pig	Gansu,Hezuo	This study
HT384		Hezuo Tibetan pig	Gansu,Hezuo	This study
HT385		Hezuo Tibetan pig	Gansu,Hezuo	This study
HT386		Hezuo Tibetan pig	Gansu,Hezuo	This study
QT001		Qinghai Tibetan pig	Qinhai	This study
QT002		Qinghai Tibetan pig	Qinhai	This study
QT003		Qinghai Tibetan pig	Qinhai	This study
QT004		Qinghai Tibetan pig	Qinhai	This study
QT005		Qinghai Tibetan pig	Qinhai	This study
QT006		Qinghai Tibetan pig	Qinhai	This study
QT007		Qinghai Tibetan pig	Qinhai	This study
QT008		Qinghai Tibetan pig	Qinhai	This study
QT009		Qinghai Tibetan pig	Qinhai	This study
QT010		Qinghai Tibetan pig	Qinhai	This study
QT011		Qinghai Tibetan pig	Qinhai	This study
QT012		Qinghai Tibetan pig	Qinhai	This study
QT013		Qinghai Tibetan pig	Qinhai	This study
QT014		Qinghai Tibetan pig	Qinhai	This study
QT015		Qinghai Tibetan pig	Qinhai	This study
QT016		Qinghai Tibetan pig	Qinhai	This study
QT017		Qinghai Tibetan pig	Qinhai	This study
QT018		Qinghai Tibetan pig	Qinhai	This study
QT019		Qinghai Tibetan pig	Qinhai	This study
QT020		Qinghai Tibetan pig	Qinhai	This study
QT021		Qinghai Tibetan pig	Qinhai	This study
QT022		Qinghai Tibetan pig	Qinhai	This study
QT023		Qinghai Tibetan pig	Qinhai	This study
QT024		Qinghai Tibetan pig	Qinhai	This study
QT025		Qinghai Tibetan pig	Qinhai	This study
QT026		Qinghai Tibetan pig	Qinhai	This study
QT027		Qinghai Tibetan pig	Qinhai	This study
QT028		Qinghai Tibetan pig	Qinhai	This study
QT029		Qinghai Tibetan pig	Qinhai	This study
QT030		Qinghai Tibetan pig	Qinhai	This study
QT031		Qinghai Tibetan pig	Qinhai	This study
QT032		Qinghai Tibetan pig	Qinhai	This study
QT033		Qinghai Tibetan pig	Qinhai	This study
QT034		Qinghai Tibetan pig	Qinhai	This study
QT035		Qinghai Tibetan pig	Qinhai	This study
QT036		Qinghai Tibetan pig	Qinhai	This study
QT037		Qinghai Tibetan pig	Qinhai	This study
QT038		Qinghai Tibetan pig	Qinhai	This study
QT039		Qinghai Tibetan pig	Qinhai	This study
QT040		Qinghai Tibetan pig	Qinhai	This study
QT041		Qinghai Tibetan pig	Qinhai	This study
QT042		Qinghai Tibetan pig	Qinhai	This study
QT043		Qinghai Tibetan pig	Qinhai	This study
QT044		Qinghai Tibetan pig	Qinhai	This study
QT045		Qinghai Tibetan pig	Qinhai	This study
QT046		Qinghai Tibetan pig	Qinhai	This study
QT047		Qinghai Tibetan pig	Qinhai	This study
QT048		Qinghai Tibetan pig	Qinhai	This study
QT049		Qinghai Tibetan pig	Qinhai	This study
QT050		Qinghai Tibetan pig	Qinhai	This study
QT051		Qinghai Tibetan pig	Qinhai	This study
QT052		Qinghai Tibetan pig	Qinhai	This study
QT053		Qinghai Tibetan pig	Qinhai	This study
QT054		Qinghai Tibetan pig	Qinhai	This study
QT055		Qinghai Tibetan pig	Qinhai	This study
QT056		Qinghai Tibetan pig	Qinhai	This study
QT057		Qinghai Tibetan pig	Qinhai	This study
QT058		Qinghai Tibetan pig	Qinhai	This study
QT059		Qinghai Tibetan pig	Qinhai	This study
QT060		Qinghai Tibetan pig	Qinhai	This study
QT061		Qinghai Tibetan pig	Qinhai	This study
QT062		Qinghai Tibetan pig	Qinhai	This study
QT063		Qinghai Tibetan pig	Qinhai	This study
QT064		Qinghai Tibetan pig	Qinhai	This study
QT065		Qinghai Tibetan pig	Qinhai	This study
QT066		Qinghai Tibetan pig	Qinhai	This study
QT067		Qinghai Tibetan pig	Qinhai	This study
QT068		Qinghai Tibetan pig	Qinhai	This study
QT069		Qinghai Tibetan pig	Qinhai	This study
QT070		Qinghai Tibetan pig	Qinhai	This study
QT071		Qinghai Tibetan pig	Qinhai	This study
QT072		Qinghai Tibetan pig	Qinhai	This study
QT073		Qinghai Tibetan pig	Qinhai	This study
QT074		Qinghai Tibetan pig	Qinhai	This study
QT075		Qinghai Tibetan pig	Qinhai	This study
QT076		Qinghai Tibetan pig	Qinhai	This study
QT077		Qinghai Tibetan pig	Qinhai	This study
QT078		Qinghai Tibetan pig	Qinhai	This study
QT079		Qinghai Tibetan pig	Qinhai	This study
QT080		Qinghai Tibetan pig	Qinhai	This study
QT081		Qinghai Tibetan pig	Qinhai	This study
QT082		Qinghai Tibetan pig	Qinhai	This study
QT083		Qinghai Tibetan pig	Qinhai	This study
QT084		Qinghai Tibetan pig	Qinhai	This study
QT085		Qinghai Tibetan pig	Qinhai	This study
QT086		Qinghai Tibetan pig	Qinhai	This study
QT087		Qinghai Tibetan pig	Qinhai	This study
QT088		Qinghai Tibetan pig	Qinhai	This study
QT089		Qinghai Tibetan pig	Qinhai	This study
QT090		Qinghai Tibetan pig	Qinhai	This study
QT091		Qinghai Tibetan pig	Qinhai	This study
QT092		Qinghai Tibetan pig	Qinhai	This study
QT093		Qinghai Tibetan pig	Qinhai	This study
QT094		Qinghai Tibetan pig	Qinhai	This study
QT095		Qinghai Tibetan pig	Qinhai	This study
QT096		Qinghai Tibetan pig	Qinhai	This study
QT097		Qinghai Tibetan pig	Qinhai	This study
QT098		Qinghai Tibetan pig	Qinhai	This study
QT099		Qinghai Tibetan pig	Qinhai	This study
QT100		Qinghai Tibetan pig	Qinhai	This study
QT101		Qinghai Tibetan pig	Qinhai	This study
QT102		Qinghai Tibetan pig	Qinhai	This study
QT103		Qinghai Tibetan pig	Qinhai	This study
QT104		Qinghai Tibetan pig	Qinhai	This study
QT105		Qinghai Tibetan pig	Qinhai	This study
QT106		Qinghai Tibetan pig	Qinhai	This study

Selected wild boar and domestic pig

Haplotype	Accession	Breed/status	Category	References
BS	AB015087	Japan	wild boar	Greger Larson et al.2005
AE	AF486859	China Xiang	domestic pig	Greger Larson et al.2005
GX	AF486862	China Tong Cheng	domestic pig	Greger Larson et al.2005
Y	AF486863	China JinHua	domestic pig	Greger Larson et al.2005
AB	AF486867	China Wuzhisan	domestic pig	Greger Larson et al.2005
T	AF136566	Sweden Large White	domestic pig	Greger Larson et al.2005
D	AY232890	Spain Spotted Black Jabugo	domestic pig	Greger Larson et al.2005
	AF304201	Italian	wild boar	Gui-Sheng Wu et al.2007
	DQ496775	MDYZ	wild boar	Gui-Sheng Wu et al.2007

Supplementary Table S2 table. The distribution of variable sites of the mtDNA D loop in Tibetan pigs.

Haplotype	Polymorphism sites<br>1111111111111111111111111111111111111111111<br>5555555555555555555555555555555555555555555<br>3333333333333333333333333333344444444444444<br>7777777888888888889999999999000000000011111<br>2345678901234567890123456789012345678901234	Population<br>Aba	 Ganzi	 Diqing	 Linzhi	 Shannan	 Changdu	 Rikeze	 Hezuo	 Qinghai	 Total
AF486859	CACGTAC-ATGCTTTTCGTCTATCTACGCTTGACCAATCTTAA										
TiH1	...A...-.................................G.				4	1	1				6
TiH2	.......-.................................G.				3						3
TiH3	.......-...........T.......................					1					1
TiH4	.......-...................................	13	81	25	160	33	18	18	40	50	438
TiH5	.......-....A.A...................G........		1								1
TiH6	.......-......A...................G........		1								1
TiH7	.......-................A.........G....C...		1								1
TiH8	.......-..........................G........		1								1
TiH9	.......-............................G......								2		2
TiH10	.......-.....................C.............				4						4
TiH11	.......-.....C..........................C..		7	2							9
TiH12	.......-.C...C..........................C..			1							1
TiH13	.......-.....C.........T................C..			6	2		1				9
TiH14	.......-..A.............................C..			1			1				2
TiH15	.......-................................C.T						1				1
TiH16	.......-................................C..			6	1		1				8
TiH17	.......-.....C.............................	1				1					2
TiH18	GC.....-...............T...................								2		2
TiH19	GC.....-...................A...............								1		1
TiH20	.......-...................A...............		2			2	3		13		20
TiH21	.......-...............T...................	19		7		14	11	4	85	7	147
TiH22	.......-..........C....T...................								1		1
TiH23	.......-T..............T...................								9		9
TiH24	.......-...............T..G..........C.....								2		2
TiH25	.......-...............T..G................								2		2
TiH26	.......-.........A.....T..G................								1		1
TiH27	..G....-...............T..G................								9		9
TiH28	..G...A-...............T..G................								1		1
TiH29	..G....-........T......T..G................								2		2
TiH30	.......-.......C.A.........................								2		2
TiH31	.......-.........A...G.....................									1	1
TiH32	.......-.........A.........................	10			6	11	15		38	13	93
TiH33	.......-........TA.........................					3	2		5		10
TiH34	.......-.........A.....T...................								1		1
TiH35	.......-........T......T...........G.C.....	2									2
TiH36	.......-........T......T.............C.....	8				3	3		4	1	19
TiH37	.......-........T....................C.....		1								1
TiH38	.......-........T.............C......C.....		1								1
TiH39	.......-...............T.............C.....			16			3		1		20
TiH40	.......-.............................C.....				5	1	1		1		8
TiH41	.......-........T...........T..............		1		3						4
TiH42	.....C.-........T..........................			2							2
TiH43	.......-........T..........................	7		11	30	8	13		9	18	96
TiH44	.......-........T......T.........G.........			1							1
TiH45	.......-........T......T.G.................			1							1
TiH46	.......-........T......T...................	10	22	89	18	10	12	2	23	10	196
TiH47	.......-........T......T................C..			6							6
TiH48	.......-........T......T..........G........		1								1
TiH49	.......-........T......T............G......			3			2				5
TiH50	.......-........T......T.....C.............								2		2
TiH51	.......-........T..............A...........									1	1
TiH52	.......-........T...G..T.......A...........									1	1
TiH53	.......-.....C..T...............G..........		2		2						4
TiH54	.......-.....C..T......T...................					1			4		5
TiH55	.......-...T....T......T...................		11		2						13
TiH56	.......-...T....T..........................				1						1
TiH57	....C..-...T..........CT..............T....					2	2		3		7
TiH58	....C..A...T..........CT..............T....									4	4
TiH59	.......-...T...........T...................								5		5
TiH60	.......-...T...............................			1							1
Total		70	133	178	241	91	90	24	268	106	1201
